# Versatile and Selective Biomolecule Pulldown with Combinatorial DNA‐Crosslinked Polymers

**DOI:** 10.1002/anie.202517600

**Published:** 2025-12-18

**Authors:** Sarah K. Speed, Krishna Gupta, Yu‐Hsuan Peng, Elisha Krieg

**Affiliations:** ^1^ Division of Polymer Biomaterials Science Leibniz Institute of Polymer Research Dresden Dresden 01069 Germany; ^2^ Faculty of Chemistry and Food Chemistry Technische Universität Dresden Dresden 01062 Germany

**Keywords:** Biomolecule pulldown, Nanotechnology, Nucleic acids, Phase separation, Programmable materials

## Abstract

Current methods for sequence‐selective biomolecule isolation suffer from high cost, off‐target effects, and limited flexibility. Here, we introduce *LASSO* (cross**L**ink‐**A**ssisted **S**equence‐**S**elective is**O**lation), a versatile platform using programmable polymer phase separation to capture biomolecules under native conditions. LASSO relies on *combinatorial crosslinker libraries*—diverse mixtures of DNA strands that collectively trigger the formation of highly swollen polymer agglomerates with near‐zero background binding. We demonstrate >80% pulldown efficiency for diverse targets, including DNA, SARS‐CoV‐2 RNA, and human thrombin. LASSO provides 8–20x higher binding capacity (4 nmol mg^−1^ polymer) than commercial microbeads. In RNA‐seq workflows, LASSO depleted ribosomal RNA with 86% efficiency, while yielding up to 7x fewer off‐target outliers versus state‐of‐the‐art magnetic beads and enzyme‐based methods. Thrombin was captured via switchable aptamers with 90% efficiency, and a gentle release mechanism allowed the subsequent isolation of 98% enzymatically active proteins from the polymer. LASSO's cost‐effectiveness ($0.96/sample versus $46–$51 for commercial kits), long‐term stability (7 + years), simple usage, and modularity position it to advance diagnostics, transcriptomics, and bionanotechnology workflows.

## Graphical abstract

We present a programmable smart polymer system for universal, sequence‐selective bioseparation under native conditions. Polymer phase separation is achieved through combinatorial DNA crosslinking, enabling the capture of DNA, RNA, or proteins in a homogeneous solution with high pulldown efficiency and near‐zero background binding. This method reduces biases in ribosomal RNA depletion for RNA‐seq, while offering long‐term stability and cost‐efficiency.

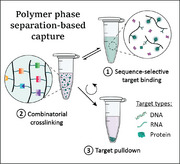

## Introduction

The separation and purification of biomolecules from complex mixtures is a fundamental process in chemistry, biology, and medical diagnostics.^[^ ^1^, ^2^ ^]^ Traditional pulldown methods use inexpensive solid‐phase resins to separate complex biomolecule mixtures based on molecular size, charge, or other features. The resins bind broad categories of biomolecules (e.g., nucleic acids) while leaving others in solution.^[^ ^3^, ^4^ ^]^ However, more specific methods are often needed to isolate biomolecules in a sequence‐selective manner. This is typically accomplished through complementary sequence hybridization (for nucleic acids) or through antibodies (for proteins). Magnetic microbeads functionalized with affinity ligands have emerged as the dominant solution for sequence‐selective capture.^[^ ^5^, ^6^, ^7^, ^8^ ^]^ After binding their target, the microbeads are readily pulled down with a magnetic field. Other popular approaches utilize enzymes to selectively degrade non‐target nucleic acid sequences.^[^ ^9^, ^10^ ^]^ The greatest drawbacks of currently available sequence‐selective methods are their relatively high cost (e.g., due to the use of enzymes or other recombinant proteins), off‐target effects (e.g., due to non‐specific adsorption on microbeads), and time‐consuming sample preparation.^[^ ^11^, ^12^, ^13^ ^]^

Over recent decades, several stimuli‐responsive or “smart” polymers have been developed as a scalable option for bioseparation. Such polymers respond to small changes in environmental conditions, such as temperature, ionic strength, pH, or electric or magnetic fields.^[^ ^14^, ^15^, ^16^, ^17^ ^]^ Specific ligands for target capture can be linked to the polymer backbone, forming an “affinity macroligand”.^[^ ^18^, ^19^, ^20^, ^21^, ^22^ ^]^ After the target is bound to this ligand, the polymer is precipitated, thereby pulling the target out of solution. We previously used this principle to develop a methanol‐responsive polymer (MeRPy) that selectively binds and isolates single‐ and double‐stranded DNA targets.^[^ ^23^, ^24^ ^]^

Several challenges limit the wider adoption of smart polymers in bioseparation (cf. Table S1): First, most reported methods rely on precipitation triggers that are impractical for complex biological samples.^[^ ^25^, ^26^, ^27^ ^]^ These triggers (e.g., temperature, pH, and organic co‐solvent) can be innocuous for some biomolecule types while causing denaturation or aggregation in others. An exception is the popular elastin‐like polypeptides (ELP), as they permit relatively mild pulldown triggers.^[^ ^28^ ^]^ However, ELPs depend on genetically fused affinity tags that must be customized for each target, confining their application to recombinant proteins. Second, polymer precipitation may be triggered inadvertently during target capture. For example, binding of nucleic acids to hybridization probes typically requires thermal annealing, which would cause premature precipitation of thermoresponsive polymers.^[^ ^17^, ^28^ ^]^ This problem can be mitigated by introducing a third component to trigger phase separation only after target binding is complete.^[^ ^29^ ^]^ Third, current systems frequently suffer from non‐specific co‐precipitation of non‐target biomolecules.^[^ ^20^, ^30^, ^31^ ^]^ For example, MeRPy pulldown enables sequence‐selective isolation of DNA,^[^ ^24^ ^]^ but fails to achieve comparable selectivity for RNA. Finally, all existing smart polymer systems lack true programmability and remain limited to narrow target types. Their recognition sites are often covalently linked to the polymer, making them difficult to exchange or adapt for new targets.

Here we report a versatile pulldown method termed LASSO (cross**L**ink‐**A**ssisted **S**equence‐**S**elective is**O**lation), which overcomes the key limitations of smart polymers in affinity precipitation. LASSO represents the first pulldown polymer capable of universal, sequence‐selective bioseparation under native conditions. It operates through a bio‐orthogonal crosslinking mechanism that controls polymer agglomeration and phase separation. A key innovation is the use of combinatorial DNA crosslinker libraries, which promote the efficient formation of sparse molecular networks from dilute polymer solutions. The polymer can be readily programmed for the capture of highly diverse target types by simply exchanging non‐covalently bound DNA ligands that encode target recognition. With LASSO, we demonstrate highly specific and efficient capture of DNA, RNA, and protein with minimal background binding, as well as the release of selected targets back into solution. As a practical demonstration, we apply LASSO to ribosomal RNA (rRNA) depletion for RNA sequencing (RNA‐seq) library preparation, achieving efficient depletion with reduced off‐target bias as compared to the two most widely used commercial rRNA depletion methods. We further show the pulldown and release of human thrombin protein under native conditions, preserving the protein's structure and enzymatic activity.

## Results and Discussion

### Material Concept

**Figure 1:**
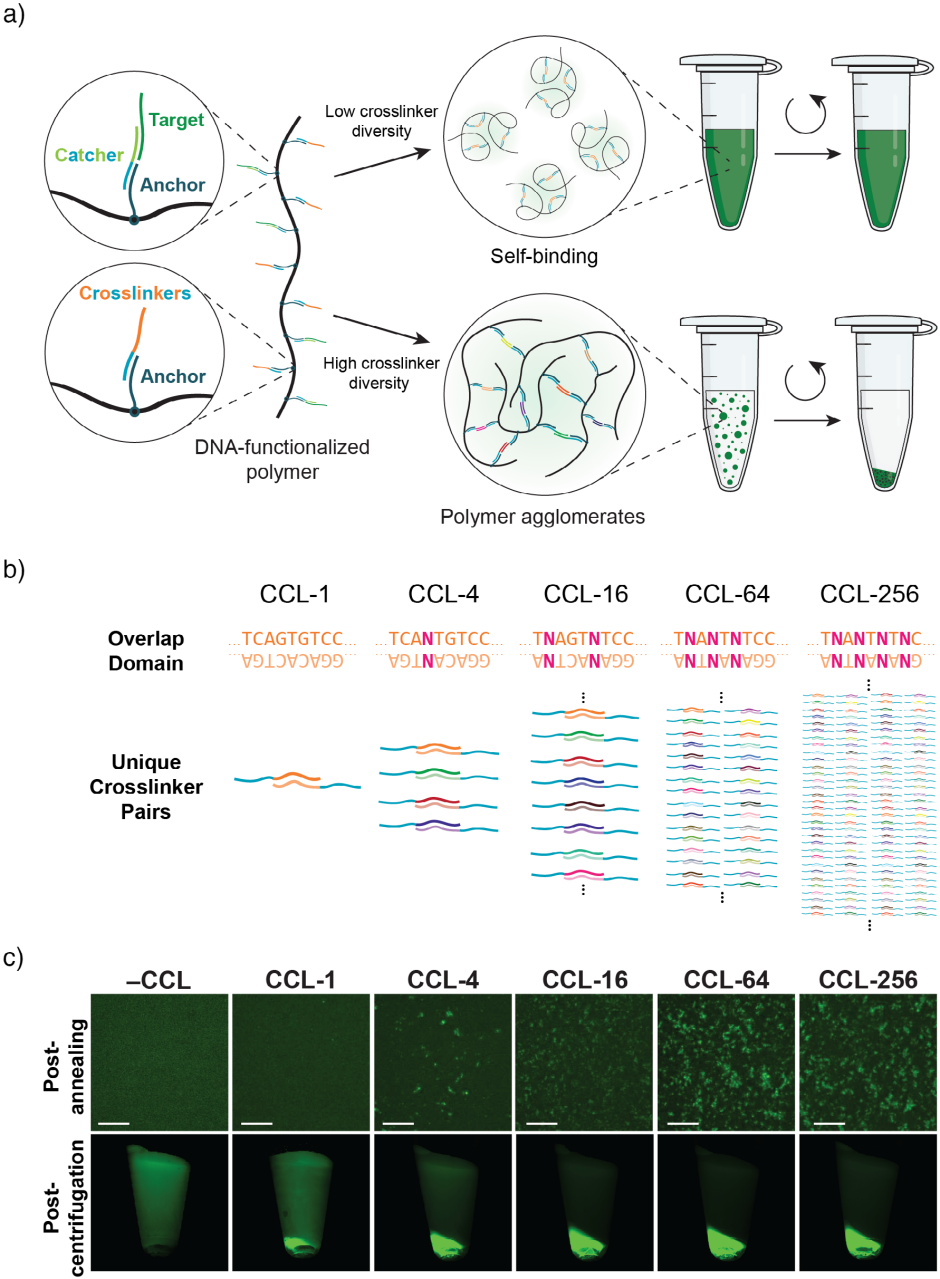
LASSO uses crosslinking‐induced phase separation for selective biomolecule pulldown under mild conditions. a) Target‐specific DNA catcher strands and DNA crosslinkers hybridize to DNA anchor strands conjugated to ultra‐high molecular weight polymer chains. A low diversity of crosslinkers leads to ineffective intra‐molecular bond formation, whereas a high diversity of crosslinkers promotes inter‐molecular crosslinking and phase separation. Target‐bound polymer agglomerates are centrifuged into a pellet, separating and encapsulating the target. b) Combinatorial crosslinker library (CCL) approach in which the overlap domain is diversified through the introduction of mixed “N” nucleotides. The complexity of the library increases with the number of N nucleotides in the sequence. c) Effect of CCL diversity on phase separation. Top row: Fluorescent microscope images of P_20_ without crosslinkers or crosslinked with different CCLs at 100x magnification and stained with SYBR Gold. Scale bar: 15 µm. Bottom row: Pulldown of a Cy5‐labeled target DNA oligonucleotide on P_20_ crosslinked with different CCLs.

LASSO relies on three components: an acrylamide‐based *polymer*,^[^ ^23^, ^24^, ^32^ ^]^ DNA oligonucleotide *catcher strands*, and DNA *crosslinkers* (Figures 1 and S1). The polymer is functionalized with DNA *anchor strands*. These serve as binding sites to program the material's phase separation and affinity characteristics with crosslinkers and catcher strands, respectively. We synthesized two variants of the polymer, **P_10_** and **P_20_**, differing in their degree of functionalization with anchor strands (see Supporting Methods 1.2; Figures S2 and S3). The catcher strands comprise a target‐specific binding domain, an adapter domain complementary to the anchor strand, and an (optional) toehold domain for triggering target release through toehold‐mediated strand displacement (TMSD) (Figure S1a,c). The binding domain consists of either a linear DNA sequence to capture DNA or RNA via hybridization or an aptamer sequence to capture a protein.

We envisioned that the addition of DNA crosslinkers would trigger the formation of large polymer agglomerates, leading to phase separation and thereby providing a gentle pulldown mechanism (Figures 1a and S1b). Such a *crosslink‐induced secondary effect affinity precipitation* is highly uncommon,^[^ ^33^, ^34^ ^]^ likely because in dilute polymer solutions, intra‐molecular binding is favored over crosslinking,^[^ ^35^, ^36^ ^]^ making polymer agglomeration vastly inefficient (Figure 1a). However, we recently discovered that this problem can be solved by using *combinatorial crosslinker libraries* (CCL)—complex mixtures of DNA strands containing many distinct recognition sites.^[^ ^36^ ^]^ Sufficient crosslinker diversity suppresses ineffective intra‐molecular bonds. We therefore followed our previous approach,^[^ ^36^ ^]^ in which the overlap domains of crosslinker pairs are diversified by introducing *mixed bases* (N) at specific positions, where N can be either of the four canonical nucleobases with roughly equal probability (Figure 1b). The number of unique crosslinker pairs in the CCL is 4^n^, where n is the number of N bases in the sequence. In addition, the adapter and overlap domains of the crosslinkers were designed to have well‐separated melting temperatures in order to ensure successive binding when cooled from 95 to 20 °C in a one‐pot annealing process (Figure S4).

We first tested how crosslinker diversity affected the phase separation of **P_20_**, using five different CCLs with *n* = 0 (“CCL‐1”), *n* = 1 (“CCL‐4”), *n* = 2 (“CCL‐16”), *n* = 3 (“CCL‐64”), and *n* = 4 (“CCL‐256”). Microscopically visible polymer agglomerates started to form at CCL‐4, with the most distinct particles appearing in the CCL‐64 sample (Figure 1c, top row). The same trend was observed in pulldown experiments using a benchtop centrifuge, where CCL‐64‐crosslinked polymers most efficiently isolated a fluorescently labeled DNA target (Figure 1c, bottom row; Figure S5, Supporting Procedure 1). We therefore chose CCL‐64 for subsequent experiments with LASSO. To further understand the phase separation process, we imaged polymers in the presence of CCL‐64 during cooling from 47 to 37 °C. Particulate structures appeared within 1 min and progressively enlarged, resulting in a fully phase‐separated state within less than 10 min (Figure S6 and Video S1).

LASSO's CCL‐64‐crosslinked polymers form a sparse molecular network that is swollen by 99.7% (w/v) water, with an estimated mesh size (ξ) of 32 nm, thus allowing efficient permeation with both small and large biomacromolecular targets (see Methods section). The absence of a sharp, solid–liquid interface and the low polarizability contrast with the medium disfavor nonspecific surface adsorption.^[^ ^37^ ^]^ These properties were expected to provide substantially increased binding capacity and selectivity as compared to solid resins or microbeads.

We optimized LASSO by testing the pulldown of DNA using different concentrations of **P_10_** and CCL‐64 (Figures S7 and S8; Supporting Note 1). The most efficient target capture was observed when using a polymer concentration of 0.05% (w/v), while 80% of available anchor strands were bound to CCL‐64 and 10% of anchors were bound to catcher strands. Under these conditions, LASSO can bind up to 2 nmol target per milligram **P_10_** and 4 nmol target per milligram **P_20_**. These optimized conditions allow for quantitative binding of catcher strands and crosslinkers to the polymer at the concentrations used in subsequent experiments (Figure S9).

### LASSO Efficiently and Specifically Captures DNA and RNA Targets

**Figure 2:**
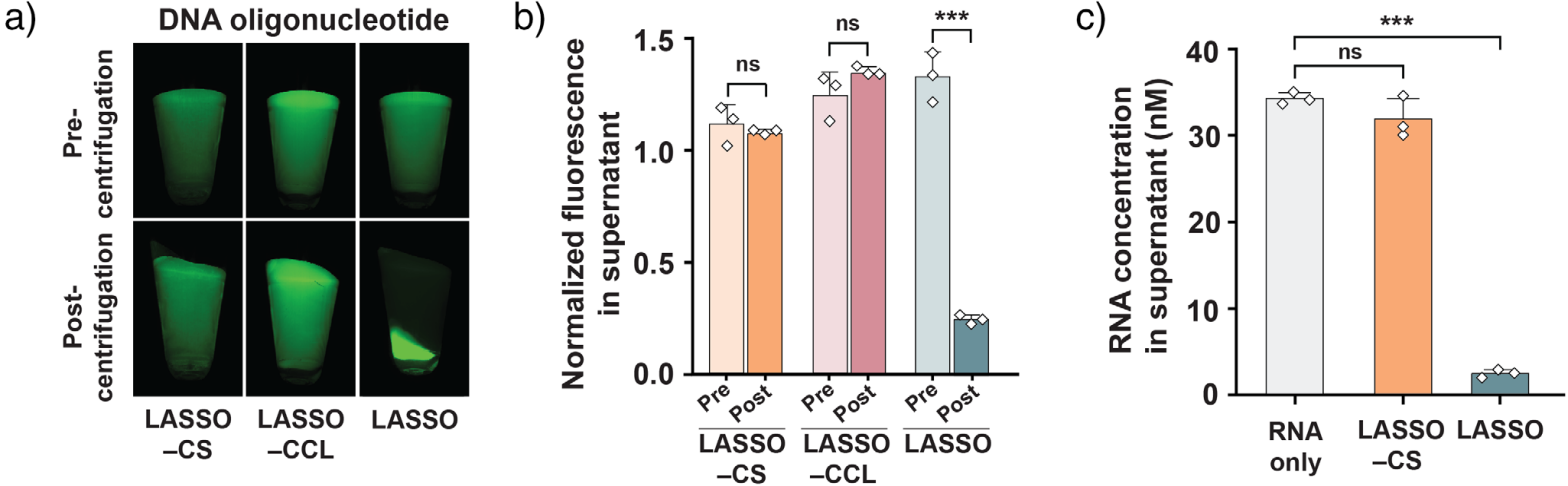
Programmable nucleic acid capture with LASSO is efficient and highly specific. a) Capture and pulldown of a fluorescent DNA oligonucleotide on P_10_ is only observed in the presence of both catcher strands (CS) and crosslinkers (CCL). b) Quantification of the DNA target remaining in the supernatant after pulldown, based on fluorescence intensity. c) Concentration of SARS‐CoV‐2 N‐gene RNA in the supernatant of the indicated samples as determined by RT‐qPCR (Figure S10). Data for panels (b) and (c) are shown as mean ± s.d. (*n* = 3 independent experiments). Statistical analysis was performed using an unpaired two‐tailed *t*‐test; ns, non‐significant (*p* > 0.05); ***p < 0.001.

To demonstrate the efficiency and specificity of the LASSO method for biomolecule capture, we used CCL‐64‐crosslinked **P_10_** to capture single‐stranded DNA and RNA (Supporting Procedure 2). A 22‐nucleotide (nt) fluorescent DNA target was captured with an average efficiency of 81.2 ± 0.1%, as measured by the amount of fluorescence remaining in the supernatant after centrifugation (Figure 2a,b, Supporting Data 2). Control samples lacking either the catcher strands or the crosslinkers showed no pulldown, highlighting the high capture specificity. Next, we tested the ability of LASSO to capture SARS‐CoV‐2 N‐gene RNA (1442 nt) to demonstrate potential application in sequestration of viral RNA for diagnostic assays. Each supernatant was subjected to RT‐qPCR to determine the amount of remaining N‐gene RNA (Figures 2c and S10; Supporting Data 2). On average, LASSO captured N‐gene RNA with an efficiency of 92.3 ± 1.4% (Figure 2c). In the absence of catcher strands, a pellet formed, but no significant target capture was observed, confirming a high binding specificity for the long RNA target.

### LASSO Enhances RNA‐seq Expression Profiles

We next explored whether LASSO can be used to enhance libraries for RNA‐seq. Most library preparation methods involve the removal of highly abundant and non‐informative transcripts, such as rRNA, to increase the sensitivity for functionally more relevant transcripts. The depletion of abundant species should ideally not affect non‐target transcripts or otherwise bias the transcriptomic profile.

We benchmarked LASSO against two of the most widely used commercial rRNA depletion kits, *riboPOOLs* and *NEBNext*. These two kits represent the two prevailing approaches for target depletion: riboPOOLs is based on magnetic bead capture, whilst NEBNext relies on enzymatic target degradation with RNase H.^[^ ^9^, ^10^, ^38^, ^39^, ^40^, ^41^ ^]^ We surmised that LASSO may offer superior specificity, since beads are prone to nonspecific interfacial adsorption,^[^ ^11^, ^13^ ^]^ and the enzyme‐based assays can inadvertently degrade transiently bound non‐target transcripts.^[^ ^12^ ^]^ Both cases can lead to undesired loss of non‐target transcripts and systematically biased transcriptomic data.

We first generated an rRNA‐specific *catcher strand library* (CSL) containing 195 distinct catcher strands that tile across the sequences of all six human rRNAs, namely, cytoplasmic rRNAs (28S, 18S, 5.8S, and 5S) and mitochondrial rRNAs (12S and 16S). Each catcher strand contains a 45–50‐nt target‐binding domain^[^ ^42^ ^]^ and a 20‐nt adapter domain (Supporting Data 1, strand IDs 22–216). The **P_20_** backbone was used instead of **P_10_** to ensure that many different catcher strands could be captured at sufficiently high individual concentrations. We then compared the performance of LASSO (Supporting Procedure 3), riboPOOLs, and NEBNext for the depletion of rRNA from human total RNA. A control sample treated with LASSO in the absence of CSL (LASSO –CSL) was also included for reference.

**Figure 3:**
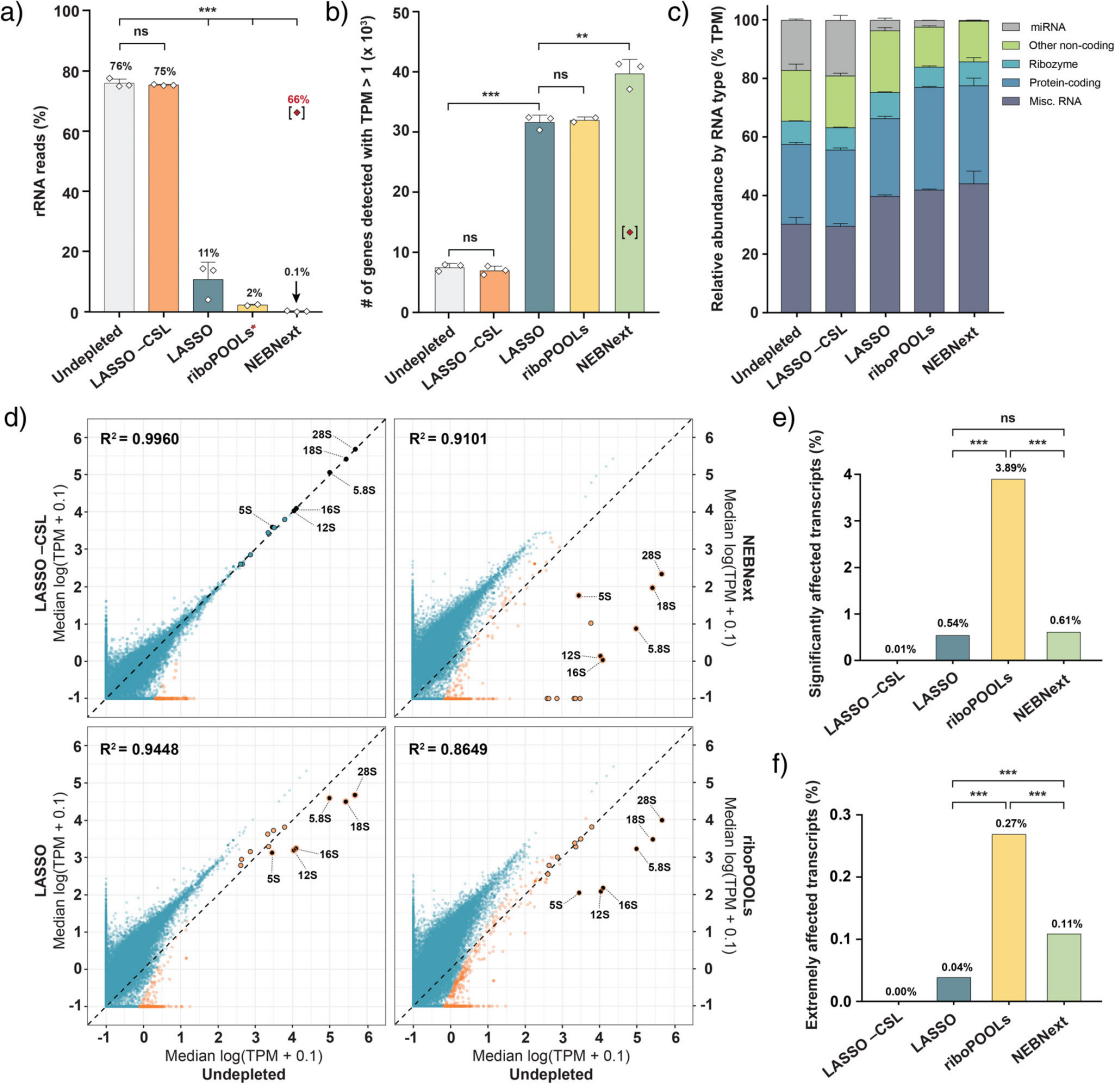
LASSO introduces fewer biases than commercial rRNA depletion kits for RNA‐seq, while providing similar increases in sequencing depth. a) Percentage of RNA‐seq reads mapped to rRNA for each condition. The red asterisk for the riboPOOLs data indicates the failure of one replicate to sequence properly due to the low final concentration. The bracketed red datapoint for the NEBNext condition corresponds to a sample prepared with an expired kit, which was excluded from data analysis. b) Total number of transcripts detected with >1 TPM for each condition. Statistical analysis for panels a and b was performed via an unpaired two‐tailed *t*‐test. c) All non‐rRNA reads were mapped to their respective Ensembl biotype annotation, normalized to transcripts per million (TPM), and grouped by type. d) Correlation analysis of expression profiles between rRNA‐depleted conditions and the undepleted condition. The six targeted rRNA transcripts are labeled. Orange data points represent outliers whose residuals lie >3 s.d. away from the linear trendline (see Methods section). Data points with black outlines indicate transcripts with high sequence similarity (>90%) to rRNA. e) Percentage of significantly affected and f) extremely affected transcripts for each method as shown in the volcano plots in Figure S15. Transcripts with padj < 0.05 and absolute log_2_ fold change >1 or padj < 0.001 and absolute log_2_ fold change >3 are considered significantly or extremely affected, respectively. Statistical analysis was performed using a two‐sided Fisher's exact test. All data in panels (a), (b), and (c) are shown as the mean ± s.d.; data in panel d are shown as the median (n = 2 independent experiments for the riboPOOLs condition; *n*  = 3 independent experiments for all other conditions). ns, non‐significant (*p* > 0.05); **p < 0.01; ***p < 0.001.

All treated samples, as well as the original total RNA, were sequenced to compare transcriptomic data before and after rRNA depletion. The proportion of total RNA reads that mapped to rRNA was 76.0 ± 1.4% before pulldown and 75.4 ± 0.2% for LASSO–CSL. When programmed with the CSL, LASSO reduced the number of rRNA reads to 10.7 ± 5.7%, corresponding to a pulldown efficiency of ∼86%. riboPOOLs and NEBNext demonstrated higher depletion efficiencies, reducing rRNA reads to 2.3 ± 0.3% and 0.1 ± 0.1%, respectively (Figure 3a, Supporting Data 2). While the newly purchased NEBNext kit showed the highest pulldown efficiency, an expired kit yielded drastically reduced rRNA depletion, with 66% of rRNA remaining in the sample (Figure 3a). This outlier was excluded from further analysis, but the strong decrease in depletion efficiency highlights the susceptibility of enzyme‐based assays to degradation and variations in performance.

For all three methods, rRNA depletion significantly increased the number of reads exceeding the threshold of one transcript per million (TPM) (Figures 3b and S11, Supporting Data 2). LASSO depletion provided a 4.3 ± 0.5‐fold increase in TPM values >1 for non‐rRNA transcripts, demonstrating a substantial enhancement of the RNA‐seq library. The increase in TPM values >1 was statistically indistinguishable from riboPOOLs (4.4 ± 0.4‐fold), but lower than for samples depleted by the newly purchased NEBNext kit (5.3 ± 0.1‐fold).

To compare undesirable off‐target depletion between the three methods, we first mapped all non‐rRNA reads in each library to the Ensembl RNA biotypes (Figures 3c and S12; Supporting Data 2). Among the three tested methods, depletion with LASSO produced the least biased biotype distribution, retaining a significantly higher percentage of micro RNAs (miRNA) and small nuclear RNAs (snRNA; included in the “other non‐coding” biotype), when compared to NEBNext (p = 0.0018 for miRNA; *p* = 0.0004 for snRNA) and riboPOOLs (*p* = 0.0034 for snRNA) (Figure 3c).

We also correlated the expression levels of individual transcripts in the depleted samples with those of the original, undepleted samples (Figures 3d and S13). Pearson correlation coefficients (R) were highest for LASSO (*R* = 0.959–0.977), followed by NEBNext (*R* = 0.934–0.955) and riboPOOLs (*R* = 0.919–0.934) (Figure S13). To highlight systematic biases arising from non‐target depletion, we marked transcript outliers in the expression plots (Figure 3d, orange data points, Figure S14; see Methods section). The significance of the outliers was further quantified by differential analysis of transcript counts (Figures 3e,f and S15; Supporting Data 2). Despite their widespread use, substantial depletion of several non‐rRNA transcripts was observed in both the NEBNext and riboPOOLs samples. riboPOOLs produced the largest number of significantly affected and extremely affected outliers (3.89% and 0.27% of transcripts, respectively). NEBNext produced fewer outliers than riboPOOLs (0.61% significant and 0.11% extreme outliers). However, several abundant non‐target transcripts were depleted by more than 3 orders of magnitude or even entirely eliminated from the library (Figures 3d and S15). LASSO produced the lowest number of outliers out of all three methods (0.54% significant and 0.04% extreme outliers) (Figures 3e,f and S15; Supporting Data 2).

Among the highly expressed transcripts (with original TPM >100), LASSO produced only seven outliers, compared with 49 outliers for riboPOOLs and 35 outliers for NEBNext (Figure 3d). All seven outliers in the LASSO samples were also affected in the commercial methods, and in some cases were completely removed in the NEBNext samples. Further analysis revealed that these seven common outliers are rRNA‐derived miRNAs and long non‐coding RNAs (lncRNA) that share >90% similarity with the targeted rRNA sequences (Figure 3d, data points with black outlines, Supporting Data 3). Among these outliers are MIR3648‐1/‐2, MIR663A/B, and MIR663AHG, which have been previously associated with oncogenesis in gastric, colon, and kidney cancers.^[^ ^43^, ^44^, ^45^ ^]^ Other highly expressed transcripts, such as RNU2‐1 and the U2 family of snRNAs, were undesirably depleted in riboPOOLs and NEBNext, but fully retained in LASSO. These RNAs are essential components of the spliceosome and are overexpressed in lung, pancreatic, and colorectal cancers, making them potential candidates as diagnostic biomarkers in serum.^[^ ^46^, ^47^ ^]^

Notably, LASSO–CSL showed no significant distortions in biotype distribution and virtually no individual transcript outliers (Figure 3c,f; Figure S15). Therefore, all instances of non‐specific pulldown in LASSO can be attributed to spurious interactions of the transcripts with the CSL. Furthermore, the comparison between two independent batches of the undepleted control sample and the LASSO–CSL sample shows no significant differences in the resulting libraries (Figure S16). Altogether, this data demonstrates that the phase separating polymer itself does not interfere with any components of the transcriptome, thus providing near‐zero background capture specificity.

### Switchable Aptamers Enable Protein Capture and Release

**Figure 4:**
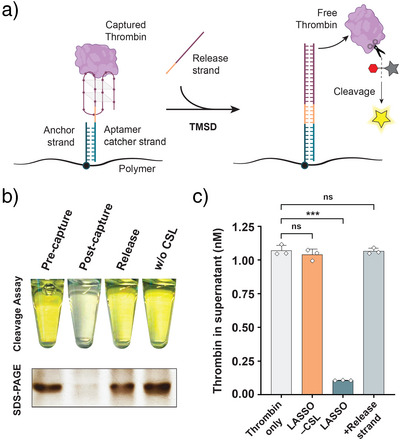
LASSO gently captures and releases thrombin through switchable aptamer binding. a) Scheme depicting the release of captured thrombin by the addition of a release strand that switches the aptamer strand from an accessible folded state to an inaccessible double‐stranded state through TMSD. b) Cleavage assay in which S‐2238 substrate changes from colorless to yellow through selective cleavage by thrombin in the supernatant. The SDS‐PAGE gel below shows the relative amounts of thrombin present in the corresponding samples above. The original tube and gel images are shown in Figure S17. c) Concentration of thrombin in the supernatant of the indicated samples as determined by absorbance measurements of the cleaved substrate. Data are shown as mean ± s.d. (*n* = 3 independent experiments). Statistical analysis was performed using an unpaired two‐tailed *t*‐test; ns, non‐significant (*p* > 0.05); ***p < 0.001.

To further demonstrate the broad utility of LASSO, we programmed **P_20_** to capture and release human thrombin with a switchable aptamer CSL (Figure 4a, Supporting Procedure 4). To this end, we created switchable versions of two previously reported thrombin aptamers, TBA and HD22,^[^ ^48^, ^49^ ^]^ by including a toehold sequence downstream of the aptamer domain of the catcher strand (Figure S1c). The addition of a suitable release strand was expected to trigger a TMSD reaction,^[^ ^50^, ^51^ ^]^ thereby switching the aptamer from a folded accessible state to a fully double‐stranded inaccessible state. This switching mechanism would release the captured native protein back into solution (Figure 4a).

To test both the capture efficiency and the activity of thrombin after release, we performed a cleavage assay with the thrombin‐specific chromogenic peptide substrate S‐2238. This experiment allowed for quantitative colorimetric analysis of thrombin activity in the supernatant before and after capture, and after its release back into the solution (Figures 4b,c and S17; Supporting Data 2). We additionally confirmed the presence of thrombin by SDS‐PAGE (Figures 4b and S17). We observed a significant decrease in thrombin concentration and activity in the supernatant after capture on LASSO, corresponding to a 90.1 ± 0.5% capture efficiency. After the addition of the release strand, 98.2 ± 1.2% of the captured thrombin was released back into the supernatant. No significant capture was observed in the absence of the aptamer CSL (Figure 4c). These results demonstrate that LASSO enables gentle and selective capture and release of active proteins in their native form.

## Conclusion

LASSO is a programmable and selective method for biomolecule pulldown. Its key feature is a novel phase separation mechanism that relies on combinatorial crosslinker libraries (CCL): mixtures of many different DNA strands that generate highly permeable polymer agglomerates that can scavenge target molecules and sediment during centrifugation. This approach complements recent advancements in phase‐separation engineering based on nucleic acids.^[^ ^52^, ^53^, ^54^ ^]^ Importantly, the entire capture and separation process occurs in free solution, without the involvement of solid–liquid interfaces. This reduces the likelihood of nonspecific surface adsorption, which is a persistent challenge for methods based on microbeads and other solid substrates.^[^ ^11^, ^13^ ^]^ The ability to capture targets in a homogeneous phase also enables very high binding capacities of up to 4 nmol per milligram polymer, while bead‐based assays typically bind 0.2–0.5 nmol per milligram substrate.^[^ ^55^ ^]^

**Figure 5:**
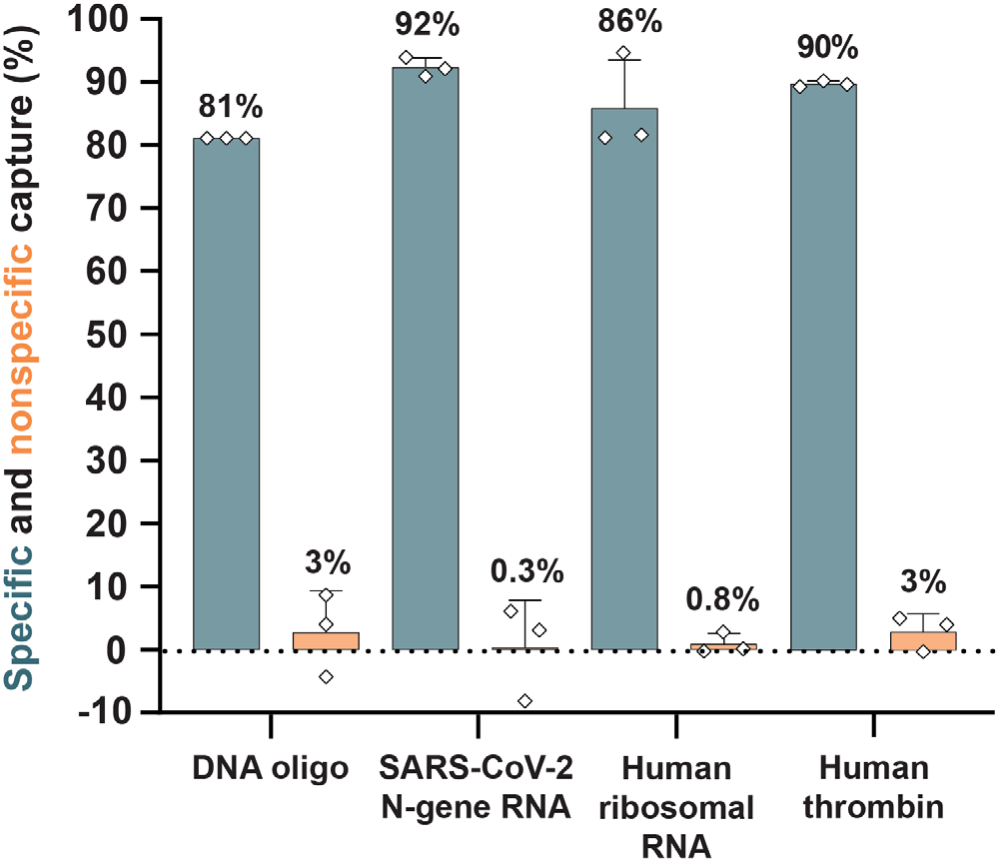
Summary of biomolecule capture on LASSO. LASSO enables highly specific and efficient capture of DNA, RNA, and protein in a programmable and target‐specific manner. Capture efficiencies were quantified by fluorescence measurements (DNA oligo), RT‐qPCR (SARS‐CoV‐2 N‐gene RNA), RNA‐seq (human ribosomal RNA), and absorbance measurements (human thrombin). Nonspecific capture was measured from samples subjected to LASSO pulldown without CSL under otherwise identical conditions. The data are shown as mean ± s.d. (*n* = 3 independent experiments).

LASSO addresses historical shortcomings of smart polymers and circumvents many drawbacks of earlier approaches in affinity precipitation (Table S1).^[^ ^21^, ^33^, ^56^ ^]^ Unlike other smart polymer systems^[^ ^33^, ^34^, ^56^ ^]^ such as the popular thermo‐responsive ELP^[^ ^28^ ^]^ and poly(N‐isopropylacrylamide),^[^ ^20^ ^]^ LASSO pulldown does not require heating‐induced precipitation, addition of chemicals, or harsh environmental changes to capture targets. Therefore, the polymer can be programmed to isolate diverse types of biomolecules sequence‐selectively and under native conditions, which is ideal for sensitive targets. We achieve consistently high capture efficiencies (81%–92%) for DNA, RNA, and protein in a one‐pot reaction that requires only basic laboratory equipment (Figure 5). The ability to release target proteins was demonstrated by using a pair of switchable aptamers, but the underlying displacement mechanism can be easily applied to any other target bound to DNA‐based catcher strands.^[^ ^24^ ^]^ The versatility of this method represents a significant advancement over previously reported MeRPy pulldown,^[^ ^23^, ^24^, ^57^ ^]^ which is applicable for DNA capture but degrades proteins and non‐specifically binds RNA (Figure S18).

Constructing and purifying sequencing libraries is one of the most immediate applications for LASSO. We depleted rRNA from RNA‐seq libraries, substantially enhancing transcriptomic profiles while demonstrating minimal off‐target bias. In particular, non‐coding RNAs, which are of interest for the study of disease biomarkers and gene regulation,^[^ ^58^, ^59^ ^]^ were more effectively retained in the LASSO‐depleted samples as compared to the commercial kits. We suspect that the biases introduced in riboPOOLs are predominantly caused by nonspecific surface adsorption, which would explain the numerous off‐target outliers that do not have substantial sequence similarity to rRNA. In contrast, biases created by the NEBNext kit are attributed to its *irreversible* depletion mechanism: if a non‐target RNA molecule binds to a probe, even transiently, it can be digested by RNase H, and is thereby irreversibly lost from the library. This explanation is consistent with the extremely strong off‐target depletion of certain miRNAs and lncRNAs that have high sequence similarity with rRNA. The distinctly different mechanism of LASSO circumvents both sources of bias: off‐target effects are limited to sequences similar to rRNA, and their depletion is weaker than the rRNA pulldown itself.

By requiring only synthetic polymer components, LASSO also provides higher stability and much lower cost when compared to conventional methods (Tables S2 and S3). Depleting rRNA from 1 µg of total RNA with LASSO costs approximately $0.96 per sample, while the two commercial kits cost $46 to $51 per sample. Storage and transportation of LASSO components do not require a cold chain, making it suitable for applications in resource‐limited settings. We further found that the DNA‐functionalized polyacrylamide backbone remains stable in the freezer for at least 7 years, without measurable reduction in binding capacity or capture efficiency (Figures S2 and S19).

Overall, LASSO enables reliable capture of diverse biomolecular targets while providing exceptional pulldown specificity and the ability to recover targets under gentle conditions. Depletion of rRNA from RNA‐seq libraries with LASSO increases sequencing depth while introducing significantly lower biases in the transcriptomic data than state‐of‐the‐art commercial kits. Our future studies will explore the use of this method for streamlining point‐of‐care diagnostic tests.^[^ ^60^, ^61^ ^]^ In particular, LASSO could be used to enrich low abundance biomarkers from clinical samples in a simple one‐step procedure.^[^ ^62^ ^]^ We anticipate that LASSO will improve preparative workflows across diverse applications, including diagnostics, bionanotechnology, next‐generation sequencing, and transcriptomics.

## Author Contributions

E.K. conceived the project. S.K.S. carried out polymer synthesis, pulldown experiments, system optimizations, and data analysis with input from E.K. and K.G. K.G. performed initial studies on polymer pulldown through DNA crosslinking. Y.‐H.P. performed the confocal microscopy imaging. S.K.S. and E.K. wrote the manuscript with input from all coauthors.

## Conflict of Interests

S.K.S., K.G., and E.K. have filed a patent relating to this technology (PCT/EP2025/057497). The remaining authors declare no conflict of interest.

## Supporting information



Supporting Information

Supporting Information

dSupporting Information

## Data Availability

All data supporting the findings of this study are provided in the article and its Supporting Information. This includes all DNA sequences (Supporting Data 1), experimental data (Supporting Data 2), and a summary of highlighted RNA‐seq outliers (Supporting Data 3). In addition, all sequencing data has been uploaded to Figshare (under identifier https://doi.org/10.6084/m9.figshare.30647552 and https://doi.org/10.6084/m9.figshare.30648857).
