# Supplementary material for: Versatile and Selective Biomolecule Pulldown with Combinatorial DNA‐Crosslinked Polymers
Source: Angew Chem Int Ed Engl. 2025 Dec 18;65(5):e17600. doi: 10.1002/anie.202517600 (PMC12851002; doi:10.1002/anie.202517600)
Supplement: Supplementary file 1 — Supporting Information [file ANIE-65-e17600-s003.docx]

**Supporting Information**

**Versatile and Selective Biomolecule Pulldown with Combinatorial DNA-Crosslinked Polymers**

[1 Supporting Methods 2](#_Toc214460398)

[1.1 Materials 2](#_Toc214460399)

[1.2 Polymer synthesis 2](#_Toc214460400)

[1.3 Polymer mesh size calculation 3](#_Toc214460401)

[1.4 LASSO capture 3](#_Toc214460402)

[1.5 SARS-CoV-2 N-gene RNA synthesis 3](#_Toc214460403)

[1.6 DNA oligonucleotide capture analysis 4](#_Toc214460404)

[1.7 SARS-CoV-2 N-gene RNA capture analysis 4](#_Toc214460405)

[1.8 RNA sequencing (RNA-seq) 4](#_Toc214460406)

[1.9 Thrombin cleavage assay 5](#_Toc214460407)

[1.10 Sodium dodecyl sulfate-polyacrylamide gel electrophoresis (SDS-PAGE) 5](#_Toc214460408)

[1.11 Nuclear magnetic resonance spectroscopy 5](#_Toc214460409)

[1.12 Polymer binding capacity assay 6](#_Toc214460410)

[1.13 Fluorescence-based analysis of crosslinker annealing 6](#_Toc214460411)

[1.14 Time-lapse fluorescence microscopy of polymer phase separation 6](#_Toc214460412)

[1.15 Optimization of the LASSO protocol 7](#_Toc214460413)

[1.16 Testing adsorption of RNA to DNA-grafted poly(acrylamide-coacrylic acid) during methanol precipitation 8](#_Toc214460414)

[1.17 Statistics and reproducibility 8](#_Toc214460415)

[2 Supporting Procedures 9](#_Toc214460416)

[3 Supporting Notes 15](#_Toc214460417)

[4 Supporting Figures 16](#_Toc214460418)

[5 Supporting Tables 35](#_Toc214460419)

[6 Supporting References 36](#_Toc214460420)

# Supporting Methods

## Materials

Solvents and reagents were purchased from commercial sources and used as received, unless otherwise specified. Water was obtained from a Milli-Q system from Merck Millipore. Molecular biology grade acrylamide (catalog number A9099) and 19:1 acrylamide/bis-acrylamide (catalog number A2917), sodium acrylate (catalog number 408220), and ammonium persulfate (APS; catalog number A3678) were purchased from Sigma-Aldrich. Methanol (ACS reagent grade; catalog number 423955000), ultrapure N,N,N′,N′-tetramethylethylenediamine (TEMED; catalog number 15524010), and SYBR™ Gold Nucleic Acid Gel Stain (catalog number S11494) were purchased from Thermo Fisher Scientific. Desalted oligonucleotides were purchased from Integrated DNA Technologies (IDT). Nitrogen gas (>99.999%) was used under inert conditions and supplied by an in-house gas generator. To ensure an inert condition, nitrogen gas was purified through a Model 1000 oxygen trap from Sigma-Aldrich (catalog number Z290246). Reagents with unreacted acrylamide groups were stored at 4 °C or −20 °C, protected from unnecessary exposure to light. The NEBNext® rRNA Depletion Kit v2 with Beads (catalog number E7405S), Monarch® Spin RNA Cleanup Kit (catalog number T2050S), and DNase I (catalog number M0303S), were purchased from New England Biolabs (NEB). The riboPOOLs rRNA depletion kit with cleanUP module was purchased from siTOOLs Biotech (catalog number dp-K012-53). Human HeLa cell total RNA was purchased from Takara Bio (catalog number 636543) and stored at −80 °C until use. Purified thrombin from human plasma (catalog number T6884) and bovine serum albumin (BSA, catalog number A2934) were purchased from Sigma-Aldrich. Chromogenix S-2238™ thrombin substrate was purchased from Diapharma (catalog number S820324). DNA ladders (catalog number SM1211) and protein ladders (catalog number LC5615) were purchased from Thermo Fisher Scientific.

## Polymer synthesis

A detailed protocol is available in ref.^[1]^. In brief, acrylamide (50 mg ml^−1^), sodium acrylate (0.5 mg ml^−1^), and acrylamide-labeled anchor strand DNA (Supporting Data 1, strand ID 1) were co-polymerized at a molar ratio of 10,000:100:x (x = 10 or 20 for **P_10_** and **P_20_**, respectively) in 1x TBE buffer (100 mM Tris, 90 mM boric acid, 1 mM EDTA, pH 8.3) to create DNA-grafted poly(acrylamide-coacrylic acid). Synthesis of **P_10_** was initiated by addition of 0.025 wt% TEMED and 0.025 wt% APS. Synthesis of **P_20_** was initiated by addition of 0.05 wt% TEMED and 0.05 wt% APS. To achieve high molecular weight and a narrow size distribution, it was necessary to carry out the reaction in high-purity nitrogen gas, which was passed through an oxygen trap on-site. The reaction was allowed to proceed overnight, resulting in a highly viscous polymer solution indicating the formation of long polymer chains. NMR spectroscopy was used to verify high conversion of the monomer (Figure S2). The solution was diluted in 9 volumes of 1x TE buffer (10 mM Tris, 1 mM EDTA, pH 8.0) and subsequently purified via methanol precipitation. The pellet was resuspended in milliQ water at 2.5% (w/v) and stored in aliquots at −20 °C. The binding capacities of **P_10_** and **P_20_** were found to be 15 nmol single-stranded DNA (ssDNA) per milligram polymer and 34 nmol ssDNA per milligram polymer, respectively (Figure S3).

## Polymer mesh size calculation

To estimate the mesh size (ξ) of the polymer network, we applied the relationship ξ ≈ ν_e_^−1/3^, where ν_e​_ is the effective crosslinker concentration in units of molecules per cubic meter, which is derived from the affine network model of rubber elasticity.^[2]^ Based on the measured volume of the pellet after centrifugation, the crosslinker concentration in the sedimented polymers agglomerates was estimated to be 50 μM (ν_e_ = 3.01 × 10^22^ molecules/m³). Assuming a uniform, isotropic 3D network, the mesh size was calculated to be ξ ≈ (3.01 × 10^22^)^−1/3^ ≈ 32 nm. This value represents the average distance between effective crosslinks within the molecular network for **P_10_**.

## LASSO capture

Detailed step-by step protocols for biomolecule capture with LASSO are described in Supporting Procedures 1–4. In brief, samples were prepared at a final concentration of 0.05% (w/v) **P_10_** or **P_20_**, mixed with up to 0.1 molar equivalents of CSL to the anchor strand, 0.8 molar equivalents of CCL-64 to the anchor strand, and 0.2–0.3 molar equivalents of the target to the CSL in a buffer condition of 150 mM NaCl and 1x TE buffer (10 mM Tris, 1 mM EDTA, pH 8.0). The samples were annealed on a C1000 Touch™ Thermal Cycler (Bio-Rad) using the following steps: (1) heating at 95 °C for 3 min, (2) instant cooling from 95 °C to 80 °C, (3) holding at 80 °C for 2 min, (4) first cooling ramp from 80 °C to 65 °C at −1.5 °C min^−1^, (5) second cooling ramp from 65 °C to 37 °C at −2.8 °C min^−1^, (6) holding at 4 °C or at 20 °C until use. The first cooling ramp facilitates binding of the adaptor domains of the crosslinkers to the anchor strands, while the second ramp allows proper binding of the overlap domains to their complementary partners. For protein capture, the protein was added after annealing and 0.1% (w/v) BSA was added to prevent non-specific protein interactions. The protein-containing mixture was then incubated with light shaking for 30 minutes at room temperature. Samples were centrifuged for 30 minutes at 17,000 xg at 4 °C or at room temperature to pellet the target-bound polymer.

## SARS-CoV-2 N-gene RNA synthesis

A T7 promoter was added to the SARS-CoV-2 N-gene sequence through PCR amplification from a 2019-nCoV_N_Positive Control plasmid (IDT, catalog number 10006625) using the Q5® Hot Start High-Fidelity 2X Master Mix (NEB, catalog number M0494S) and SAR-CoV-2 N-gene T7 primers (Supporting Data 1, strand IDs 15 and 16).^[3]^ The PCR product was purified with a DNA Clean & Concentrator spin column kit (Zymo Research, catalog number D4033) and the concentration was quantified by UV/Vis absorbance data using an Implen NanoPhotometer® P360. SARS-CoV-2 N-gene RNA was synthesized from the purified DNA product using a HiScribe® T7 High Yield RNA Synthesis Kit (NEB, catalog number E2040S). Remaining DNA was digested by addition of 20 U ml^−1^ DNase I (NEB, catalog number M0303S). EDTA was added to a final concentration of 5 mM, and DNase I was subsequently inactivated by incubation at 75 °C for 10 minutes. The final RNA product was purified using a Monarch® Spin RNA Cleanup Kit (NEB, catalog number T2050S). RNA quality and concentration were measured on a Bioanalyzer 2100 capillary electrophoresis system (Agilent) using the Agilent RNA 6000 Nano Kit (catalog number 5067-1511).

## DNA oligonucleotide capture analysis

Following capture of fluorescent DNA oligonucleotide on LASSO (Supporting Procedure 2), fluorescence images of Eppendorf tubes (in the Cy5 channel; excitation at 635 nm) were recorded before and after centrifugation on a Typhoon FLA 9500 scanner (GE Healthcare Life Sciences) at a 50 µm pixel size with the accompanying software (v1.0). Supernatant samples before and after centrifugation were quantified in a Tecan Spark® microplate reader (Tecan) by Cy5 dye fluorescence (excitation at 649 nm; emission at 697 nm).

## SARS-CoV-2 N-gene RNA capture analysis

Following capture of SARS-CoV-2 N-gene RNA on LASSO (Supporting Procedure 2), supernatant samples were diluted 1:5000 in nuclease-free H_2_O supplemented with 1 U μl^−1^ RNase inhibitor (NEB, catalog number M0307S). RNA remaining in the supernatant was amplified via RT-qPCR using the Luna® Universal One-Step RT-qPCR kit (NEB, catalog number E3005S) and SARS-CoV-2 N-gene RNA primers (Supporting Data 1, strand IDs 20 and 21). A standard curve was prepared from purified SARS-CoV-2 N-gene RNA. The samples were thermocycled on a CFX96 C1000 Touch™ Real Time Thermal Cycler (Bio-Rad). Data were analyzed on Bio-Rad CFX Maestro software (v4.1.2433.1219).

## RNA sequencing (RNA-seq)

*RNA-seq library preparation and sequencing:* Human HeLa cell total RNA (Takara Bio, catalog number 636543) was subjected to rRNA depletion with i) LASSO (Supporting Procedure 3), ii) the riboPOOLs rRNA depletion kit with cleanUP module (siTOOLs Biotech, catalog number dp-K012-53), and iii) the fresh or expired NEBNext rRNA Depletion Kit v2 (NEB, catalog number E7400S). The expired NEBNext rRNA Depletion Kit v2 was 14 months past the stated expiration date. The rRNA-specific CSL was adapted from ref.^[4]^ for use with LASSO. The procedures for each of the methods were carried out as independent triplicates, but one of the riboPOOLs replicates failed to pass quality control prior to sequencing, due to low final RNA concentration. NaCl was removed from the LASSO-depleted sample using a Monarch® Spin RNA Cleanup Kit (NEB, catalog number T2050S), then further treated with 57 U ml^−1^ DNase I (NEB, catalog number M0303S) to digest any remaining oligonucleotides. EDTA was added to a final concentration of 5 mM and the final RNA library was purified using a Monarch® Spin RNA Cleanup Kit. The commercially-depleted samples were purified according to manufacturer’s instructions using the included SPRI beads. The concentration of purified RNA samples was quantified with the QuantiFluor® RNA System (Promega, catalog number E3310) in a Tecan Spark® microplate reader (Tecan). Non-treated total RNA was submitted for sequencing as an undepleted control. Final libraries were subjected to 150-bp paired-end sequencing on the Illumina NovaSeq System (Eurofins Genomics). Each of the sequencing data contained ≥ 4.7 million reads (Supporting Data 2).

*Data analysis*: Quality control, read trimming, and pseudo-alignment with Kallisto^[5]^ were performed within the nf-core/rnaseq pipeline (v3.14.0)^[6]^ to produce TPM values. The source *Homo sapiens* genome GRCh38 release 112 was acquired from Ensembl.^[7]^ To correct for incomplete rRNA annotation in the source genome, a gene annotation file acquired from the UCSC Genome Browser^[8]^ for GRCh38 was filtered for rRNA repeat annotations (see https://github.com/zxl124/rRNA_gtfs) and concatenated to the source file. Percentage of raw rRNA reads was quantified using Ribodetector (v0.3.1).^[9]^ RNA biotypes were manually annotated to the Kallisto TPM output using the biotype entries from Ensembl for GRCh38 to determine relative abundances per RNA type. The “other non-coding” category includes artifact, lncRNA, scaRNA, scRNA, snoRNA, snRNA, sRNA, TEC (“to be experimentally confirmed”), tRNA, vault RNA, and gene and pseudogene biotypes. The TPM expression plots and the heatmap of the Pearson correlation matrix were generated in R (v4.4.1), using ggplot2 and pheatmap packages. Outliers in the TPM expression plots were calculated in R (v4.4.1) as follows. The standard deviations of the LASSO –CSL log(TPM) values as a function of the undepleted log(TPM) values were determined by assuming expected values of x = y. These standard deviations were assumed to reflect the expected spread in RNA-seq data due to sample handling as well as the limitations of sequencing depth, especially at low read numbers. These standard deviation values were plotted against the undepleted log(TPM) values and an exponential model was fit to the data to obtain continuous values of expected standard deviation for all undepleted log(TPM) values (Figure S14). For each TPM expression plot, a linear model was fit to the data and the expected standard deviations from the linear fit were calculated with the exponential model. Residuals greater than 3 standard deviations were marked as outliers. Differential transcript detection was assessed using DESeq2^[10]^ and plotted in volcano plots showing statistically significant (padj < 0.05 and absolute log₂ fold change > 1) and extremely statistically significant (padj < 0.001 and absolute log₂ fold change > 3) transcripts.

## Thrombin cleavage assay

Following the capture and release of thrombin on LASSO (Supporting Procedure 4), the concentration of thrombin in the supernatant was determined by absorbance change produced from the cleavage of the chromogenic thrombin substrate Chromogenix S-2238™ (Diapharma, catalog number S820324) as follows. A standard curve was prepared from purified thrombin (Sigma-Aldrich, catalog number T6884). To each experimental and standard curve sample, 125 μM of Chromogenix S-2238™ was added, and absorbance change at 405 nm was monitored for 20 minutes at 37 °C in a Tecan Spark® microplate reader (Tecan). The kinetic curve for each sample was reduced to the mean slope of OD min^-1^ and plotted on the standard curve to determine the concentration of thrombin in each sample.

## Sodium dodecyl sulfate-polyacrylamide gel electrophoresis (SDS-PAGE)

SDS-PAGE gels were prepared using a 30% (w/w) acrylamide/bis-acrylamide stock (37.5:1; Bio-Rad, catalog number 1610158). The supernatant samples following thrombin capture were denatured at 95 °C for 5 minutes in 1x SDS loading dye (62.5 mM Tris-HCl, 10% (v/v) glycerol, 2% (w/v) SDS, 0.01% (w/v) bromophenol blue, 5% (v/v) DTT, pH 6.8) and loaded into a 12.5% SDS-PAGE gel. The gel was run in 0.5x TBE buffer (50 mM Tris, 45 mM boric acid, 0.5 mM EDTA, pH 8.3) at 100V on a XCell SureLock Mini-Cell Electrophoresis System using a Consort™ EV265 Electrophoresis power supply (Thermo Fisher Scientific). The gel was stained with the Pierce™ Silver Stain Kit (Thermo Fisher Scientific, catalog number 24612).

## Nuclear magnetic resonance spectroscopy

Nuclear magnetic resonance (NMR) spectroscopy was performed on samples of polymers prepared in D_2_O. ^1^H NMR spectra were recorded at 30–32 °C on a 500 MHz spectrometer (Bruker) with a 2 second acquisition time and 32 transients. Chemical shifts (δ) are reported in parts per million (ppm) downfield from tetramethyl silane (TMS). The ^1^H NMR shifts are relative to the residual hydrogen peak of D_2_O (4.79 ppm).

## Polymer binding capacity assay

### Anchor strand quantification

The binding capacity of **P_10_** and **P_20_** and the stability of **P_10_** over long-term storage were determined by polyacrylamide gel electrophoresis (PAGE). Samples were prepared in 1x TE buffer (10 mM Tris, 1 mM EDTA, pH 8.0) with a final concentration of 150 mM NaCl. To test the binding capacity, either 0.0025% (w/v) of **P_10_** (maximum 0.5 µM anchor strands) or 0.00125% (w/v) of **P_20_** (maximum 0.5 µM anchor strands) was used. To test the stability of **P_10_**, 0.0033% (w/v) of a **P_10_** stock (maximum 0.5 µM anchor strands) that had been stored at −20 °C for seven years was used. The original concentration of anchor strands after synthesis was quantified as 75 µM for the 0.5% (w/v) **P_10_** stock. The binding efficiency test target strand (Supporting Data 1, strand ID 2) was added at a series equivalent from 0.2x–2.0x the amount of anchor strands for all samples. The samples were annealed according to the LASSO protocol (Supporting Procedure 1). Native polyacrylamide gels were prepared using a 40% (w/w) acrylamide/bis-acrylamide (19:1) stock. The annealed samples were loaded into a 15% native polyacrylamide gel and run in 0.5x TBE buffer (50 mM Tris, 45 mM boric acid, 0.5 mM EDTA, pH 8.3) at 100V on a XCell SureLock Mini-Cell Electrophoresis System using a Consort™ EV265 Electrophoresis power supply (Thermo Fisher Scientific). Gels were stained with SYBR™ Gold. Gel scans (using a blue laser excitation at 473 nm) were recorded on a Typhoon FLA 9500 scanner (GE Healthcare Life Sciences) at a 50 µm/pixel resolution with the accompanying software (v1.0).

### Catcher strand and crosslinker quantification

The binding efficiency of the catcher strands and crosslinkers on the polymer was determined by PAGE. A sample was prepared with 0.05% (w/v) **P_20_** (maximum 20 µM anchor strands), 80% of the anchor strand concentration of CCL-64 (Supporting Data 1, strand IDs 9 and 10), and either 150 nM, 500 nM, or 1 µM of CS (Supporting Data 1, strand ID 2) in 1x TE buffer and 150 mM NaCl. Control samples were prepared with only CS at 1 µM, CCL-64 at 16 µM, or 0.05% w/v **P_20_**. The samples were annealed according to the LASSO protocol (Supporting Procedure 1) and run on polyacrylamide gels as detailed in Section 1.12.1.

## Fluorescence-based analysis of crosslinker annealing

A sample was prepared with 0.05% (w/v) **P_20_**, 80% of the anchor strand concentration of CCL-64 (Supporting Data 1, strand IDs 9 and 10), and ), and 1x dsGreen® double-stranded DNA-binding dye (Lumiprobe) in 1x TE buffer and 150 mM NaCl. A second sample was prepared in the same manner without **P_20_.** Both samples were annealed according to the LASSO protocol (Supporting Procedure 1). The fluorescence was monitored over the annealing process in a CFX96 C1000 Touch™ Real Time Thermal Cycler (Bio-Rad).

## Time-lapse fluorescence microscopy of polymer phase separation

A sample was prepared with 0.05% (w/v) **P_20_** and 80% of the anchor strand concentration of CCL-64 (Supporting Data 1, strand IDs 9 and 10) in 1x TE buffer and 150 mM NaCl. DNA in the sample was stained with SYBR™ Gold for visualization. The sample was heated to 95 °C for 3 minutes, then immediately transferred to a 96-well plate on an Andor Dragonfly confocal microscope (Oxford Instruments). A Z-stack of 50 µm was acquired at 100x magnification every 5 seconds.

## Optimization of the LASSO protocol

Fluorescence images of all Eppendorf tubes (in the Cy5 channel; excitation at 635 nm) were recorded before and after centrifugation on a Typhoon FLA 9500 scanner (GE Healthcare Life Sciences) at a 50 µm pixel size with the accompanying software (v1.0). Image analysis of fluorescence was performed in Fiji (v1.54m). Capture efficiency was determined by comparing the fluorescence intensity in the supernatant to a DNA-only control sample after centrifugation.

### Confocal microscopy

Samples were prepared with 0.05% (w/v) **P_20_** and 80% of the anchor strand concentration of each combinatorial crosslinker library (CCL) containing 0 (uncrosslinked control), 1, 4, 16, 64 or 256 unique crosslinkers (Supporting Data 1, strand IDs 3 – 12) in 1x TE buffer and 150 mM NaCl. The samples were annealed according to the LASSO protocol (Supporting Procedure 1). DNA in the sample was stained with SYBR™ Gold for visualization. Confocal images were acquired at 100x magnification on an Andor Dragonfly confocal microscope (Oxford Instruments).

### Crosslinker diversity

The number of unique crosslinkers in each combinatorial crosslinker library (CCL) was tested for its effect on the phase separation of the polymer and capture efficiency on LASSO. Samples were prepared with 0.05% (w/v) **P_20_**, 80% of the anchor strand concentration of each combinatorial crosslinker library (CCL) containing 0 (uncrosslinked control), 1, 4, 16, 64 or 256 unique crosslinkers (Supporting Data 1, strand IDs 3 – 12), 150 nM catcher strand (Supporting Data 1, strand ID 14), and 50 nM fluorescent ssDNA oligonucleotide target (Supporting Data 1, strand ID 13) in 1x TE buffer and 150 mM NaCl. The samples were annealed and centrifuged according to the LASSO protocol (Supporting Procedure 1).

### Polymer concentration

The polymer concentration was tested for its effect on the phase separation of the polymer and capture efficiency on LASSO. Samples were prepared with 0.01–1% (w/v) **P_10_**, 90% of the anchor strand concentration of CCL-64 (Supporting Data 1, strand IDs 9 and 10), 150 nM catcher strand (Supporting Data 1, strand ID 14), and 50 nM fluorescent ssDNA oligonucleotide target (Supporting Data 1, strand ID 13) in 1x TE buffer and 150 mM NaCl. The samples were annealed and centrifuged according to the LASSO protocol (Supporting Procedure 1).

### Crosslinker concentration

The concentration of crosslinkers was tested for its effect on the phase separation of the polymer and capture efficiency on LASSO. Samples were prepared with 0.05% (w/v) **P_10_**, 10%–100% of the anchor strand concentration of CCL-64 (Supporting Data 1, strand IDs 9 and 10), 150 nM catcher strand (Supporting Data 1, strand ID 14), and 50 nM fluorescent ssDNA oligonucleotide target (Supporting Data 1, strand ID 13) in 1x TE buffer and 150 mM NaCl. The samples were annealed and centrifuged according to the LASSO protocol (Supporting Procedure 1).

## Testing adsorption of RNA to DNA-grafted poly(acrylamide-coacrylic acid) during methanol precipitation

Cytoplasmic RNA was mixed with DNA-functionalized polymers (RNA + Polymer) or with an equivalent volume of 1x TE buffer (RNA only) with varying pH and NaCl concentrations and subjected to methanol precipitation as described in ref. [^1^]. Absorbance readings at 230, 260, and 280 nm were taken in a spectrophotometer to determine RNA concentration in the supernatant.

## Statistics and reproducibility

Sample sizes and statistical tests are noted in the figure captions. Illumina sequencing was performed with three independent replicates for the LASSO, NEBNext, and *riboPOOLs* samples. One *riboPOOLs* library failed quality control due to insufficient final RNA concentration, reducing the number of *riboPOOLs* replicates to two. All other assays were performed with three independent replicates for each sample. No data were excluded from the analysis. All experiments were performed multiple times and showed consistent results, as reported in the manuscript.

# Supporting Procedures

**Supporting Procedure 1: General LASSO capture and release**

1. **Reagents**
   - 0.5% (w/v) DNA-functionalized polymer with 100 µM (**P_10_**) or 200 µM (**P_20_**) maximum anchor strand DNA (Supporting Data 1, strand ID 1) in 1x TE buffer
   - 10x Hybridization buffer (**HB**): 100 mM Tris, 10 mM EDTA, 1.5 M NaCl, pH 8.0
   - **H_2_O**
   - Catcher strand library DNA (**CSL**, mixed in equimolar ratio) in 1x TE buffer
   - 100 µM combinatorial crosslinker library with 64 unique overlap sequences (**CCL-64**, mixed in equimolar ratio) in 1x TE buffer (Supporting Data 1, strand IDs 9 and 10)
   - Release strand library DNA (**RSL**, mixed in equimolar ratio) in 1x TE buffer
   - Biomolecule **target** in buffer of choice (e.g. H_2_O, TE buffer, PBS buffer)
2. **Target capture**
3. To capture desired target (ssDNA, RNA, or protein) select the appropriate CSL and target and mix the following components together:

| **Component** | **Final Concentration** |
| --- | --- |
| P_10_ or P_20_ | 0.05% w/v (max. 10 µM or 20 µM anchor stands) |
| CSL | Up to 0.1 molar equivalents of anchor strands |
| CCL-64 | 0.8 molar equivalents of anchor strands |
| Target* | 0.3 molar equivalents of CSL |
| HB | To 1x concentration |
| H_2_O | Up to final desired volume |

*Target can be added before annealing if capturing nucleic acids, or after annealing if capturing temperature-sensitive molecules (e.g. proteins)

1. Vortex thoroughly, then anneal as follows:

(1) Heat to 95 °C for 3 min,

(2) Instant cool from 95 °C to 80 °C,

(3) Hold at 80 °C for 2 min,

(4) Cool from 80 °C to 65 °C at −1.5 °C min^−1^,

(5) Cool from 65 °C to 37 °C at −2.8 °C min^−1^,

(6) Hold at preferred temperature (e.g. 20 °C or 4 °C) until use.

1. If capturing target after annealing, add target and bind for 30 minutes with light shaking at preferred temperature.
2. Centrifuge at 17,000 xg for 30 minutes at preferred temperature.
3. The supernatant is then retrieved if target depletion is desired; optionally, the target can be released from the polymer pellet.
4. **Target release**
   1. Remove supernatant, leaving polymer pellet intact at bottom of tube.
   2. Optionally, wash polymer pellet 3x with 1x TE buffer + 150 mM NaCl by gently adding the buffer without disturbing the pellet, incubating for 30 seconds, then removing the same volume of buffer.
   3. Add 1.5x molar excess of RSL over the CSL concentration and resuspend the pellet with vigorous pipetting.
   4. Incubate for at least two hours with light shaking at preferred temperature.
   5. Centrifuge at 17,000 xg for 30 minutes at preferred temperature.
   6. The supernatant containing the released target is retrieved.

**Supporting Procedure 2: LASSO ssDNA/RNA capture**

1. **Reagents**
   - 0.5% (w/v) DNA-functionalized polymer with 100 µM (**P_10_**) maximum anchor strand DNA (Supporting Data 1, strand ID 1)
   - 10x Hybridization buffer (**HB**): 100 mM Tris, 10 mM EDTA, 1.5 M NaCl, pH 8.0
   - **H_2_O**
   - 5 µM catcher strand library DNA (**CSL**, mixed in equimolar ratio) in 1x TE buffer (Supporting Data 1, strand ID 14 for DNA, strand IDs 18 and 19 for RNA)
   - 100 µM combinatorial crosslinker library with 64 unique overlap sequences (**CCL-64**, mixed in equimolar ratio) in 1x TE buffer (Supporting Data 1, strand IDs 9 and 10)
   - 0.5 µM **target** single-stranded DNA or RNA in 1x TE buffer (Supporting Data 1, strand ID 13 for DNA, strand ID 17 for RNA)
2. **Nucleic acid capture**
   - 1. To capture specific target nucleic acids (ssDNA or RNA), select the appropriate CSL and target and mix the following components together:

| **Component** | **Volume (µL)** | **Final Concentration** |
| --- | --- | --- |
| P_10_ | 5 | 0.05% w/v (10 µM anchor strands) |
| CSL | 1.5 | 150 nM |
| CCL-64 | 4 | 8 µM |
| Target | 5 | 50 nM |
| HB | 5 | 1x |
| H_2_O | 29.5 |  |
| Total | 50 |  |

- - 1. Vortex thoroughly, then anneal as follows:

(1) Heat to 95 °C for 3 min,

(2) Instant cool from 95 °C to 80 °C,

(3) Hold at 80 °C for 2 min,

(4) Cool from 80 °C to 65 °C at −1.5 °C min^−1^,

(5) Cool from 65 °C to 37 °C at −2.8 °C min^−1^,

(6) Hold at 20 °C until use.

- - 1. Centrifuge samples at 17,000 xg for 30 minutes at room temperature.

**Supporting Procedure 3: LASSO rRNA depletion**

1. **Reagents**
   - 0.5% (w/v) DNA-functionalized polymer with 200 µM (**P_20_**) maximum anchor strand DNA (Supporting Data 1, strand ID 1)
   - 10x Hybridization buffer (**HB**): 100 mM Tris, 10 mM EDTA, 1.5 M NaCl, pH 8.0
   - Nuclease-free **H_2_O**
   - 0.5 M **EDTA** pH 8.0
   - ~100 µM catcher strand library DNA (**CSL**) in nuclease-free H_2_O (Supporting Data 1, mix of 0.45 µM each of strand IDs 22 – 210 and 2.25 µM each of strand IDs 211 – 216)
   - 100 µM combinatorial crosslinker library with 64 unique overlap sequences (**CCL-64**, mixed in equimolar ratio) in 1x TE buffer (Supporting Data 1, strand IDs 9 and 10)
   - 1 µg/µL human HeLa cell **total RNA** in H_2_O
   - DNase I (NEB, catalog number M0303S)
   - 10x DNase I buffer
   - Monarch® Spin RNA Cleanup Kit (NEB, catalog number T2050S)
2. **rRNA capture**
   - 1. To deplete rRNA, mix the following components together on ice:

| **Component** | **Volume (µL)** | **Final Concentration** |
| --- | --- | --- |
| P_20_ | 25 | 0.05% w/v (20 µM anchor strands) |
| CSL | 3.3 | 1.32 µM, 5000 ng |
| CCL-64 | 40 | 16 µM |
| Total RNA | 1 | ~100 nM, 1000 ng |
| HB | 25 | 1x |
| H_2_O | 155.7 |  |
| Total | 250 |  |

- - 1. Vortex thoroughly, then anneal as follows in a pre-heated thermocycler:

(1) Heat to 95 °C for 3 min,

(2) Instant cool from 95 °C to 80 °C,

(3) Hold at 80 °C for 2 min,

(4) Cool from 80 °C to 65 °C at −1.5 °C min^−1^,

(5) Cool from 65 °C to 37 °C at −2.8 °C min^−1^,

(6) Hold at 4 °C until use.

- - 1. Centrifuge samples at 17,000 xg for 30 minutes at 4 °C.
    2. Transfer 230 µL of supernatant to a clean tube on ice.

1. **RNA purification**
   - - 1. Purify the rRNA-depleted RNA library using the Monarch® Spin RNA Cleanup Kit, eluting into a final volume of 30 µL H_2_O.
       2. To degrade any remaining oligonucleotides, mix the following components together on ice:

| **Component** | **Volume (µL)** |
| --- | --- |
| rRNA-depleted RNA library | 30 |
| DNase I | 1 |
| 10x DNase I buffer | 3.5 |
| H_2_O | 0.5 |
| Total | 35 |

- - - 1. Incubate at 37 °C for 10 minutes.
      2. Add EDTA to a final concentration of 5 mM and purify the final RNA library using the Monarch® Spin RNA Cleanup Kit.

**Supporting Procedure 4: LASSO thrombin capture and release**

1. **Reagents**
   - 0.5% (w/v) DNA-functionalized polymer with 200 µM (**P_20_**) maximum anchor strand DNA (Supporting Data 1, strand ID 1)
   - 10x Hybridization buffer (**HB**): 100 mM Tris, 10 mM EDTA, 1.5 M NaCl, pH 8.0
   - **1% BSA buffer**: 10 mM Tris, 1 mM EDTA, 150 mM NaCl, 1% (w/v) bovine serum albumin (BSA), pH 8.0
   - **0.1% BSA buffer**: 10 mM Tris, 1 mM EDTA, 150 mM NaCl, 0.1% (w/v) BSA, pH 8.0
   - **H_2_O**
   - 10 µM catcher strand library DNA (**CSL**, mixed in equimolar ratio) in 1x TE buffer (Supporting Data 1, strand IDs 229 and 231)
   - 100 µM combinatorial crosslinker library with 64 unique overlap sequences (**CCL-64**, mixed in equimolar ratio) in 1x TE buffer (Supporting Data 1, strand IDs 9 and 10)
   - 50 µM release strand library DNA (**RSL**, mixed in equimolar ratio) in 1x TE buffer (Supporting Data 1, strand IDs 230 and 232)
   - 0.6 µM human thrombin protein **target** in 1% BSA buffer
2. **Thrombin capture**
3. To capture thrombin, mix the following components together:

| **Component** | **Volume (µL)** | **Final Concentration** |
| --- | --- | --- |
| P_20_ | 5 | 0.05% w/v (20 µM anchor strands) |
| CSL | 2.5 | 500 nM |
| CCL-64 | 8 | 16 µM |
| HB | 4.5 | 1x |
| H_2_O | 25 |  |
| Total | 45 |  |

1. Vortex thoroughly, then anneal as follows:

(1) Heat to 95 °C for 3 min,

(2) Instant cool from 95 °C to 80 °C,

(3) Hold at 80 °C for 2 min,

(4) Cool from 80 °C to 65 °C at −1.5 °C min^−1^,

(5) Cool from 65 °C to 37 °C at −2.8 °C min^−1^,

(6) Hold at 20 °C until use.

1. Add 5 µL of 0.6 µM thrombin (in 1% BSA buffer) to bring final volume to 50 µL.
2. Incubate sample for 30 minutes with light shaking at room temperature.
3. Centrifuge at 17,000 xg for 30 minutes at room temperature.
4. **Thrombin release**
5. Remove supernatant, leaving polymer pellet intact at bottom of tube.
6. Wash polymer pellet 3x with 200 µL of 0.1% BSA buffer by gently adding the buffer without disturbing the pellet, incubating for 30 seconds, then removing the same volume of buffer.
7. Add 5 µL of RSL and 0.1% BSA buffer to a final volume of 50 µL. Resuspend the pellet with vigorous pipetting.
8. Incubate for two hours with light shaking at room temperature.
9. Centrifuge at 17,000 xg for 30 minutes at room temperature.
10. The supernatant containing the released thrombin is retrieved for analysis.

# Supporting Notes

**Supporting Note 1: Optimization of polymer concentration**

The concentration of the polymer is vital for the formation of individual polymer agglomerates that are dense enough to properly pellet and efficiently capture the target. If the concentration is higher than the critical gelation concentration of 0.2% (w/v),^[11]^ the crosslinked polymers will form an extended gel that cannot be easily compressed into a compact pellet by centrifugation. However, if the concentration is too low, the polymer chains do not efficiently cross-link and target capture is reduced. We found that a polymer concentration of 0.05% (w/v) exhibits the highest capture efficiency of a fluorescent DNA oligonucleotide target while forming a sufficiently compact pellet (Figure S7).

# Supporting Figures


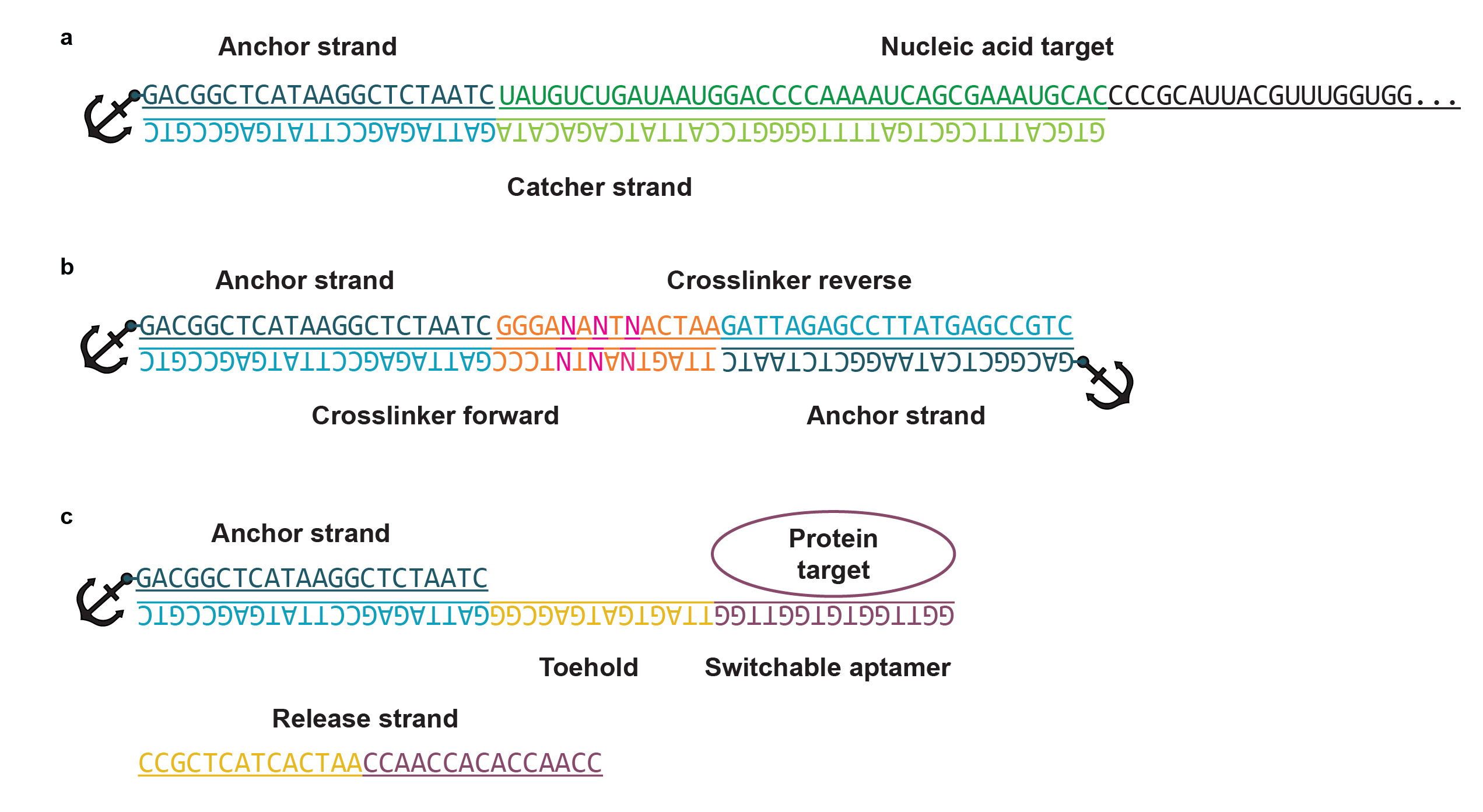


**Figure S1. Schematic representation of possible DNA complexes on LASSO. a)** Capture of nucleic acids on a catcher strand containing a target-specific binding region and a region complementary to the polymer-bound anchor strand. **b)** Crosslinking between polymer chains with a combinatorial crosslinker library (CCL). Mixed N bases in the overlap are colored in magenta. **c)** Capture of a protein target on a switchable aptamer catcher strand. The target can be released via toehold-mediated strand displacement (TMSD) through the addition of a release strand. Anchor strands are conjugated to the polymer (depicted as anchor symbols).


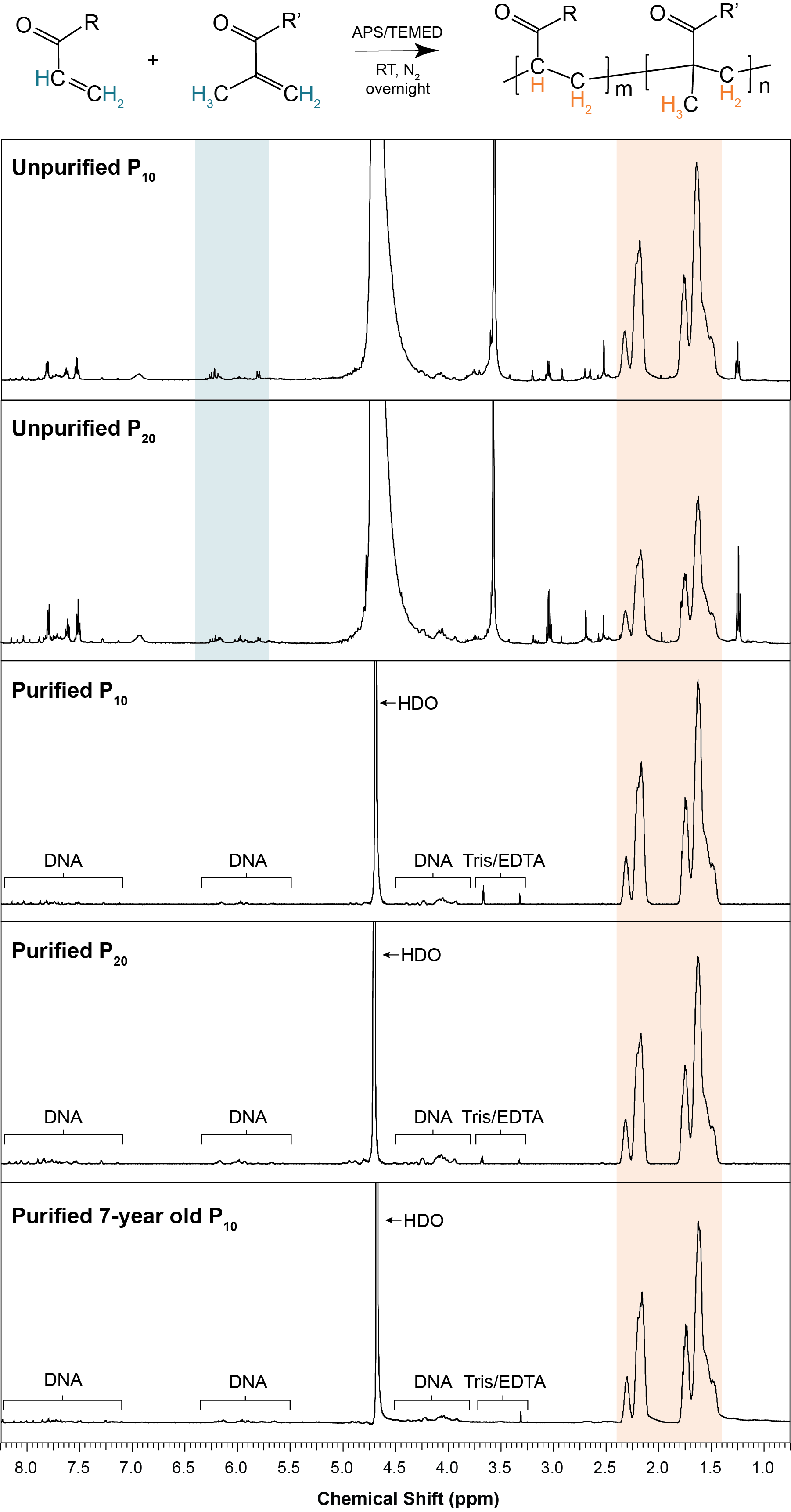


**Figure S2.** **^1^H-NMR spectra of P_10_ and P_20_ in D_2_O before and after methanol purification.**

The conversion percentage in the unpurified samples is determined by measuring the ratio of free residual acrylamide monomer protons (δ ~5.7–6.4; blue) to polymer backbone protons (δ ~1.4–2.4; orange). **P_10_** showed 99% conversion; **P_20_** showed 98% conversion. R = -NH_2_ or -OH; R' = -NH-oligonucleotides.


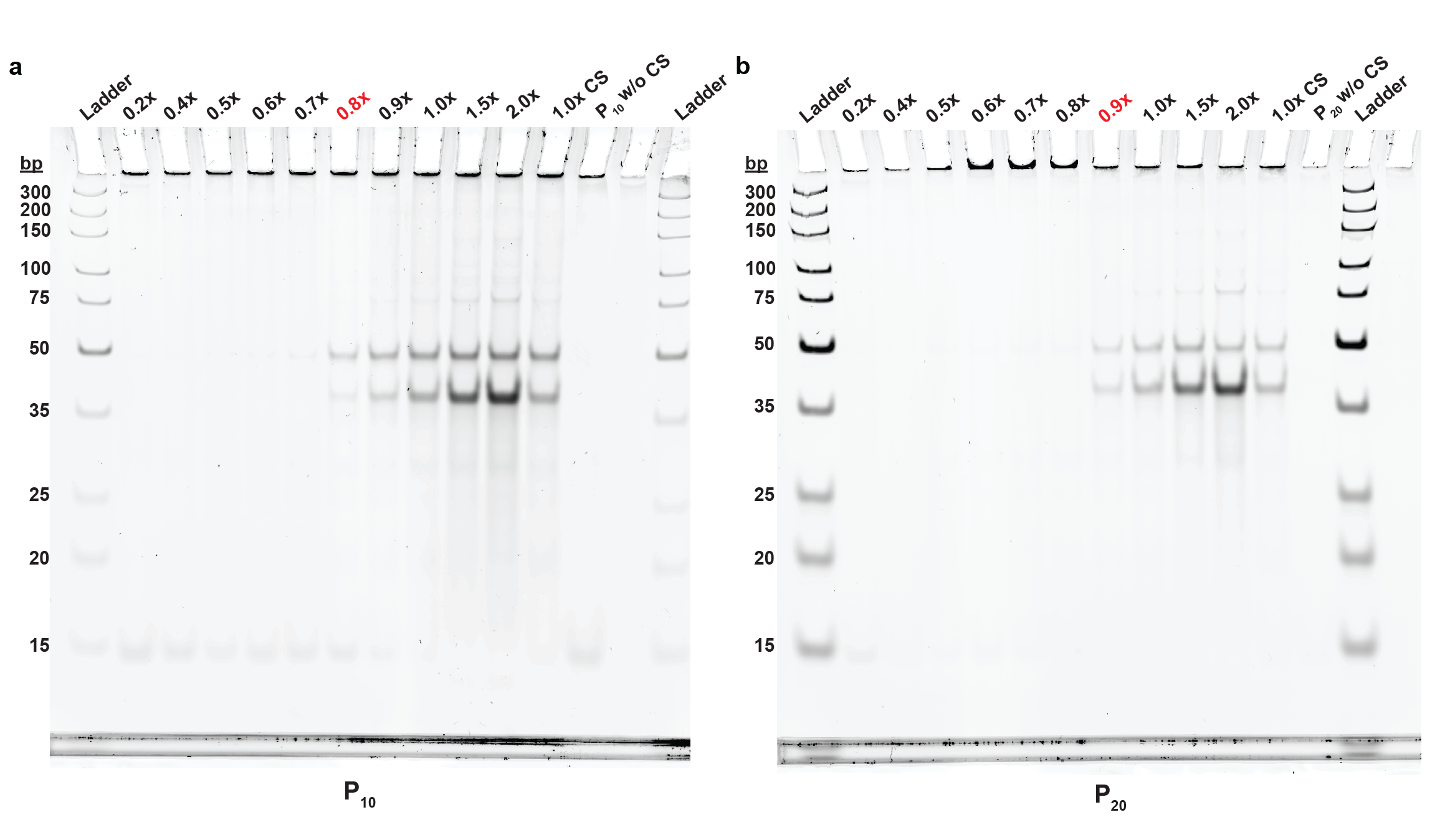


**Figure S3.** **DNA binding capacity test on P_10_ and P_20_.** The polymer was mixed with catcher strands (CS) complementary to the anchor strand at a concentration range from 0.2x to 2.0x, where 1x is defined as 100% of the maximum binding capacity. **a)** **P_10_** saturated with CS at 0.8x, indicating the available concentration of anchor strands was approximately 80 µM in a 0.5% (w/v) solution. **b)** **P_20_** saturated with CS at 0.9x, indicating the available concentration of anchor strands was approximately 170 µM in a 0.5% (w/v) solution.


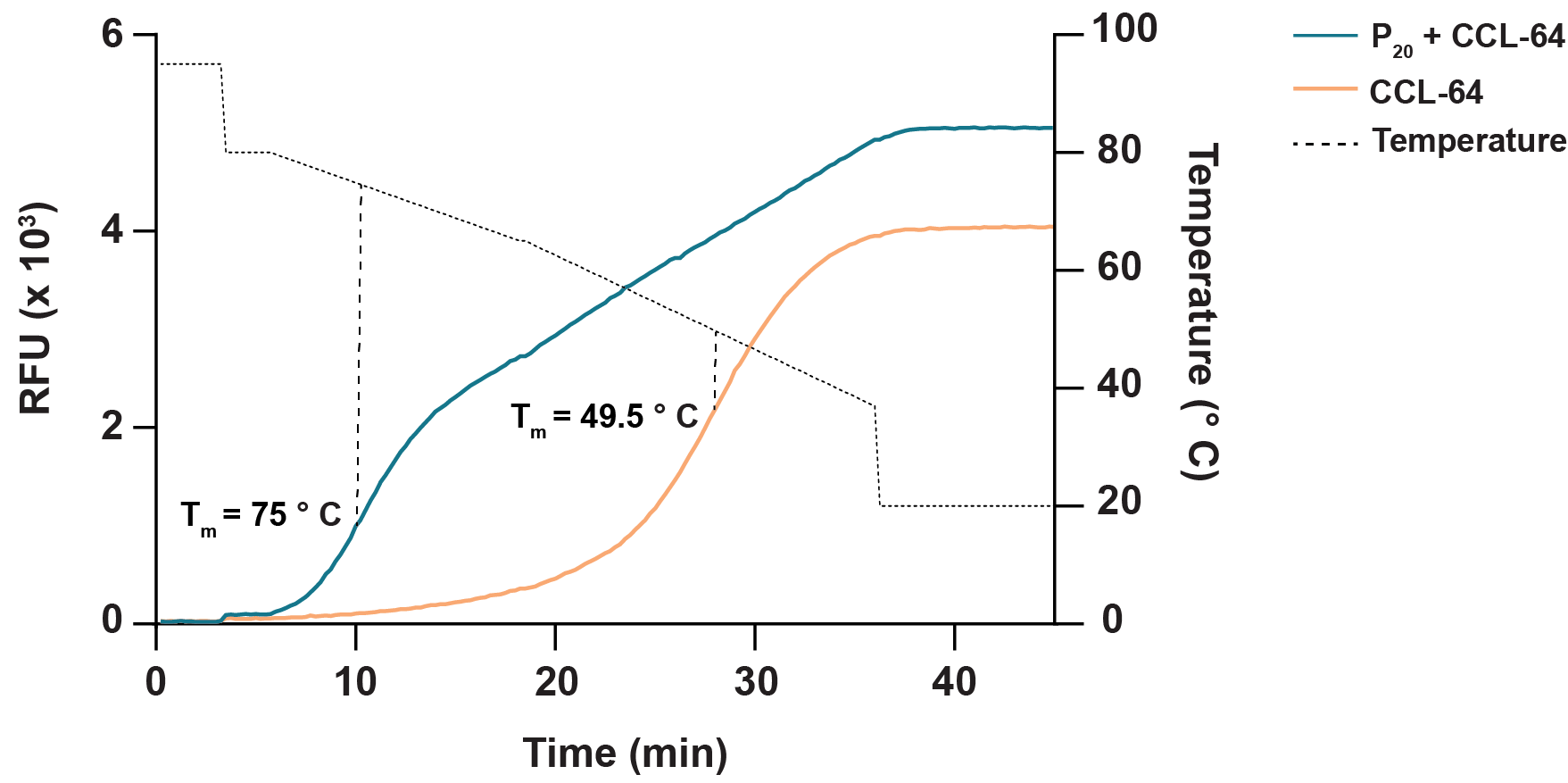


**Figure S4. Successive binding of adapter and overlap domains during annealing.** Fluorescence of a dsDNA-specific dye over time during the LASSO annealing protocol. A fluorescence increase indicates the formation of double-stranded DNA as the samples are cooled from 95 °C to 20 °C. The adapter domains of the crosslinkers bind first to the anchor strands on the polymer at 75 °C (blue trace), followed by the binding of the crosslinker overlap domains at 49.5 °C (orange trace).


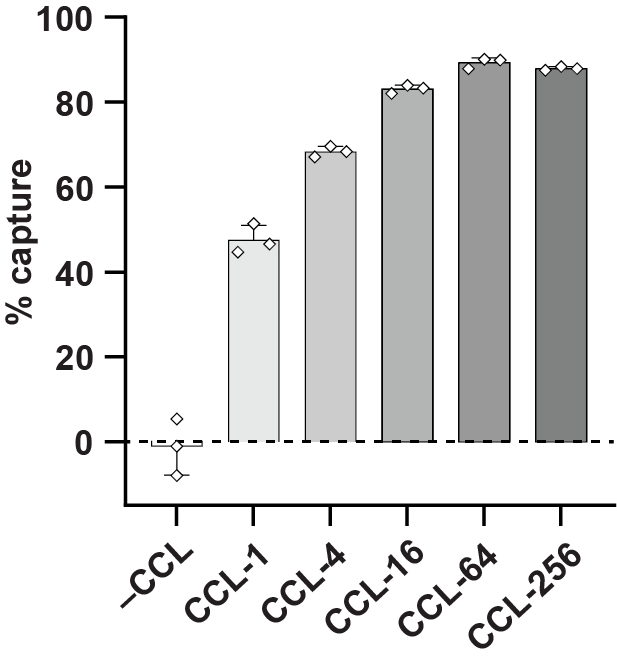


**Figure S5. Capture efficiency with different CCL diversities.** Quantification of the capture efficiency was performed via the fluorescence of a Cy5-labeled oligonucleotide target in the supernatant before and after pulldown.


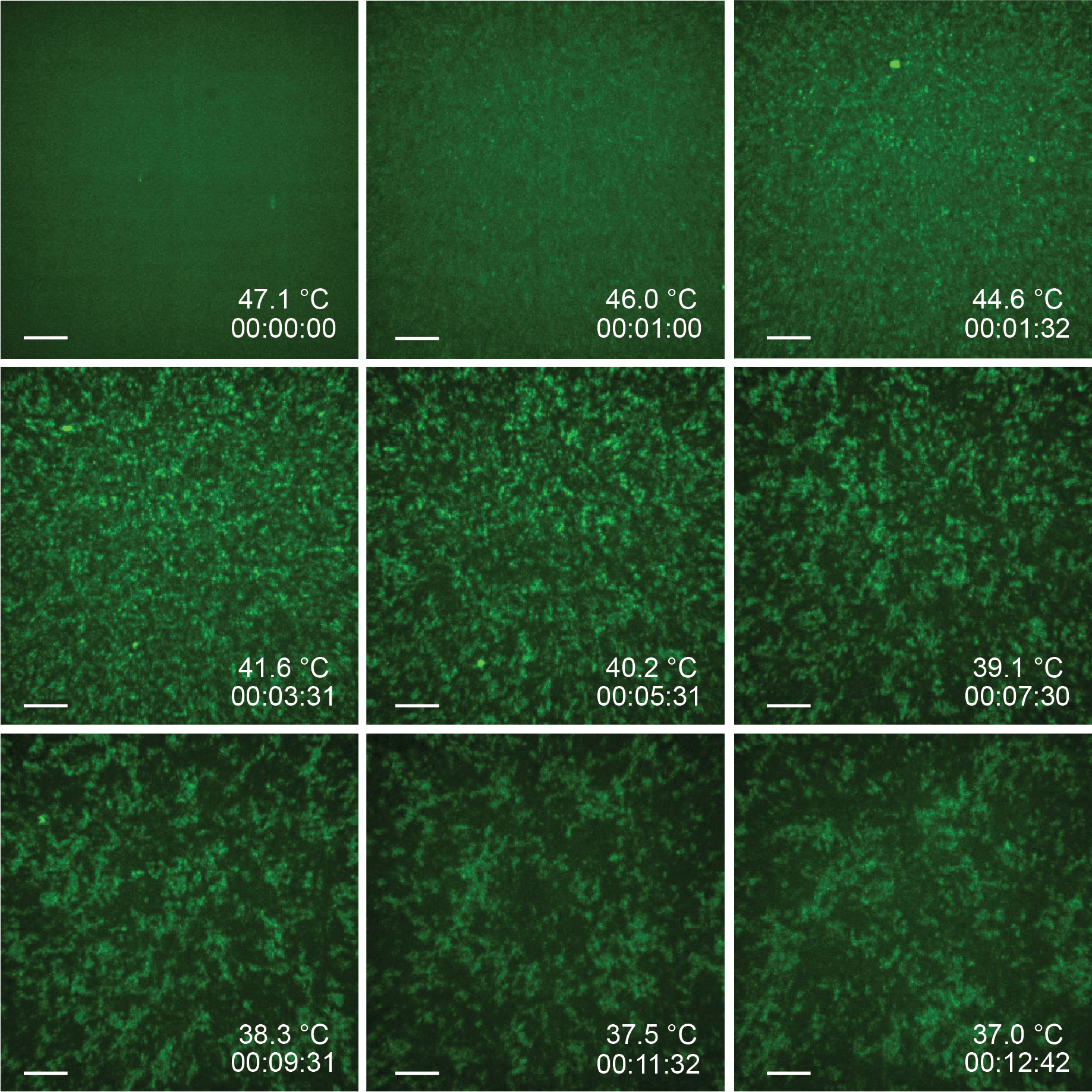


**Figure S6. Time-lapse of polymer phase separation in the presence of CCL-64.** The polymer was stained with SYBR Gold. The selected stills are taken from Video S1.

Scale bar: 15 µm. Time stamps are displayed in the format hh:mm:ss.


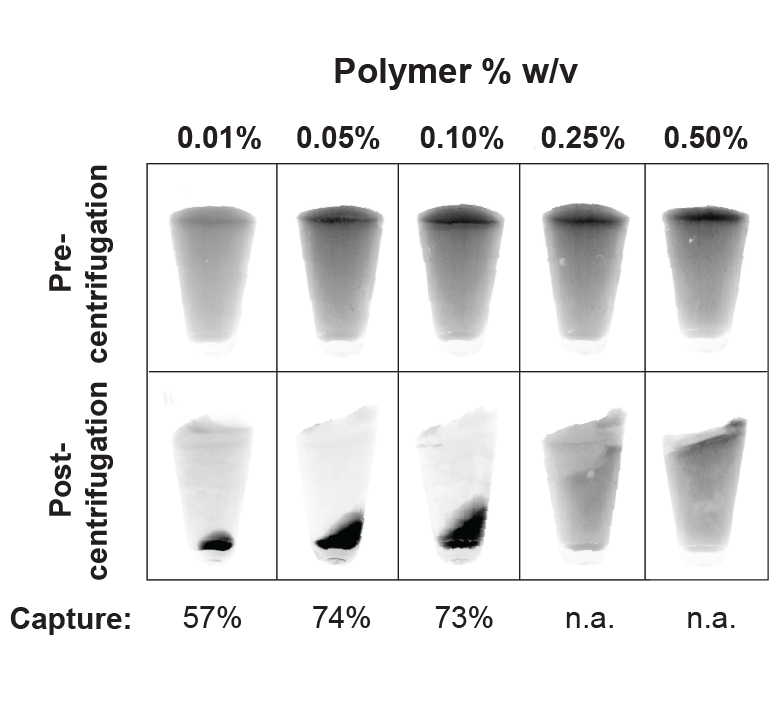


**Figure S7. Optimization of polymer concentration.** Capture of a fluorescent single-stranded DNA oligonucleotide on CCL-64-crosslinked **P_10_** at different concentrations reveals the highest capture efficiency at 0.05% (w/v). Concentrations higher than the critical gelation concentration of 0.2% (w/v) start to form an extended polymer gel that cannot be pelleted.


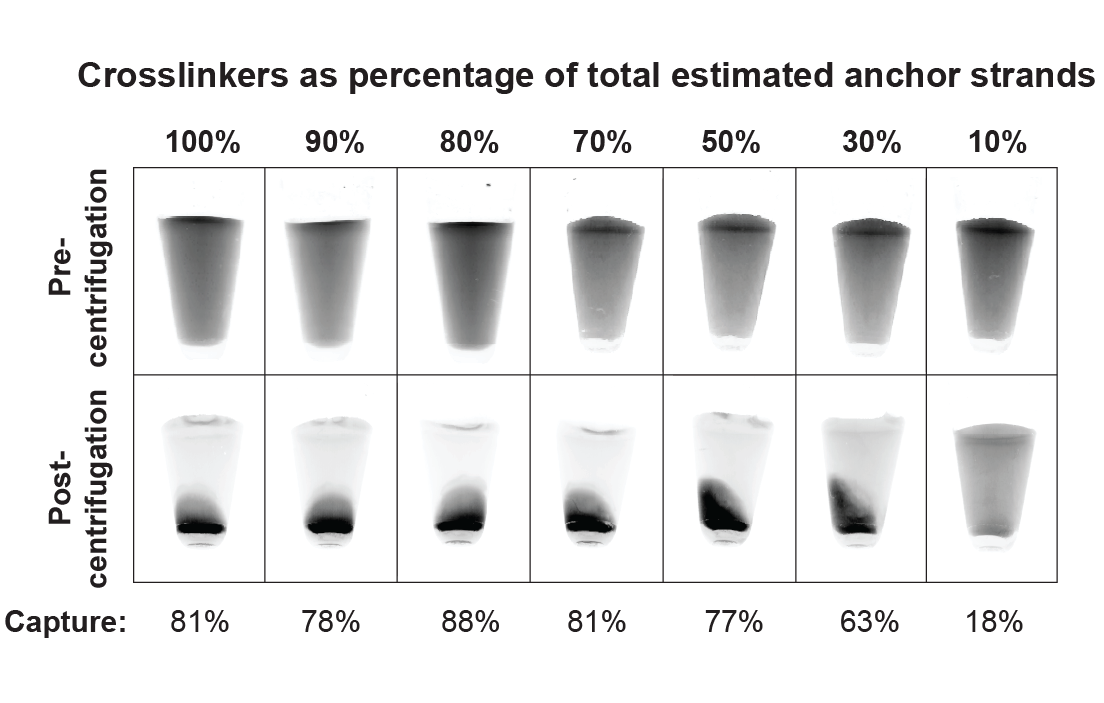


**Figure S8. Optimization of crosslinker concentration.** Capture of a fluorescent single-stranded DNA oligonucleotide on 0.05% (w/v) **P_10_** at different CCL-64 concentrations reveals the highest efficiency of capture at 80% of the total estimated anchor strand concentration.


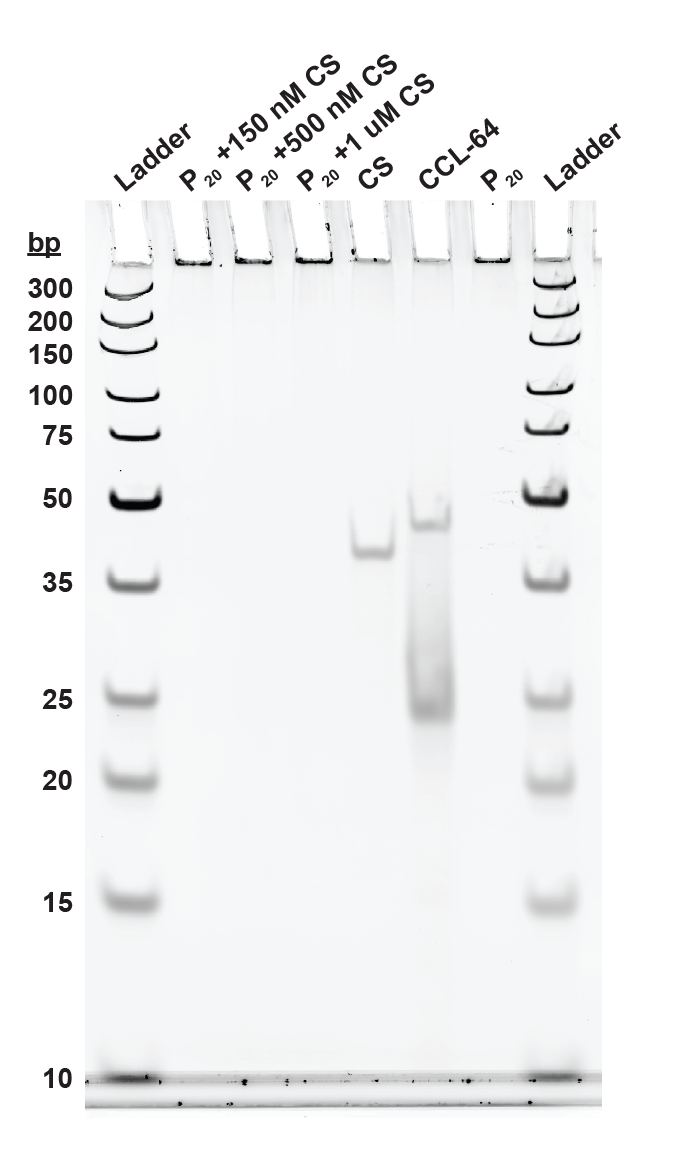


**Figure S9. Catcher strand and crosslinker binding test on P_20_.** Native PAGE of samples containing different combinations of LASSO components. Capture of catcher strands (“CS”) and crosslinkers (“CCL-64”) on 0.05% (w/v) **P_20_** show 100% capture efficiency at the concentrations used in subsequent experiments. The two bands in the CCL-64 control lane indicate the monomer (lower band) and the dimer (upper band). Bands corresponding to the polymer and polymer-bound targets remain close to the wells.


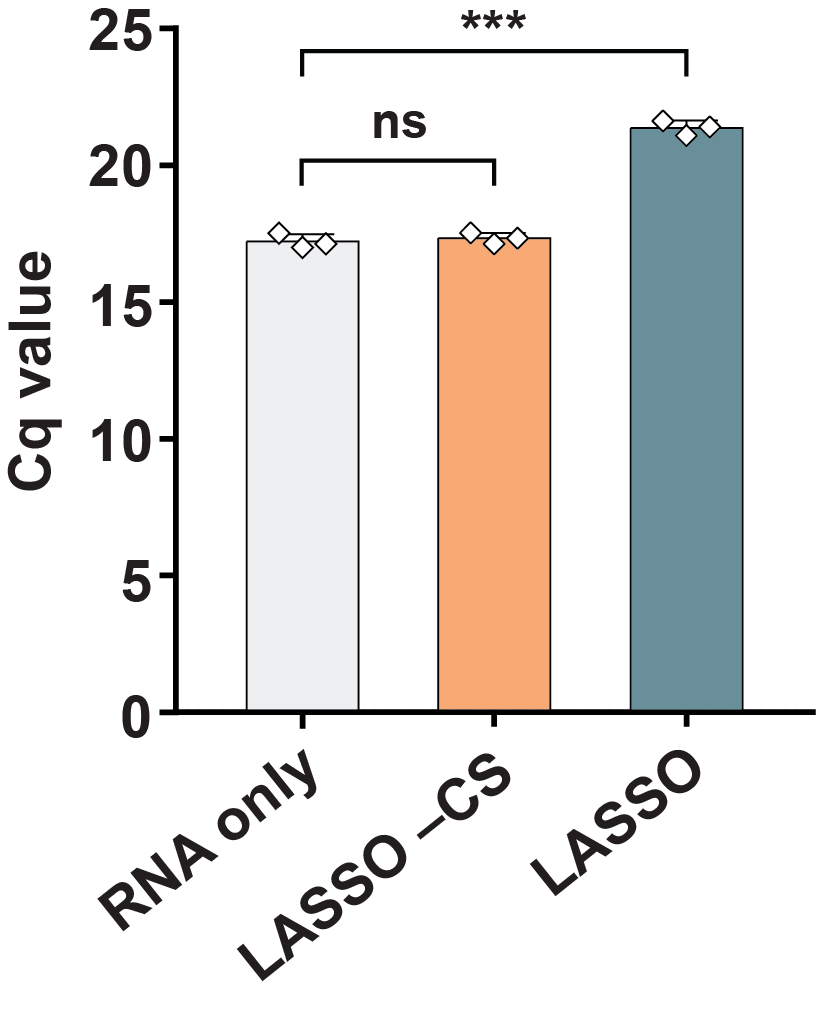


**Figure S10. Cq values for RT-qPCR of SARS-CoV-2 N-gene RNA after depletion with LASSO.** Data are shown as mean ± s.d. (n = 3 independent experiments). Statistical analysis was performed using an unpaired two-tailed t-test; ns, non-significant; ***p < 0.001.


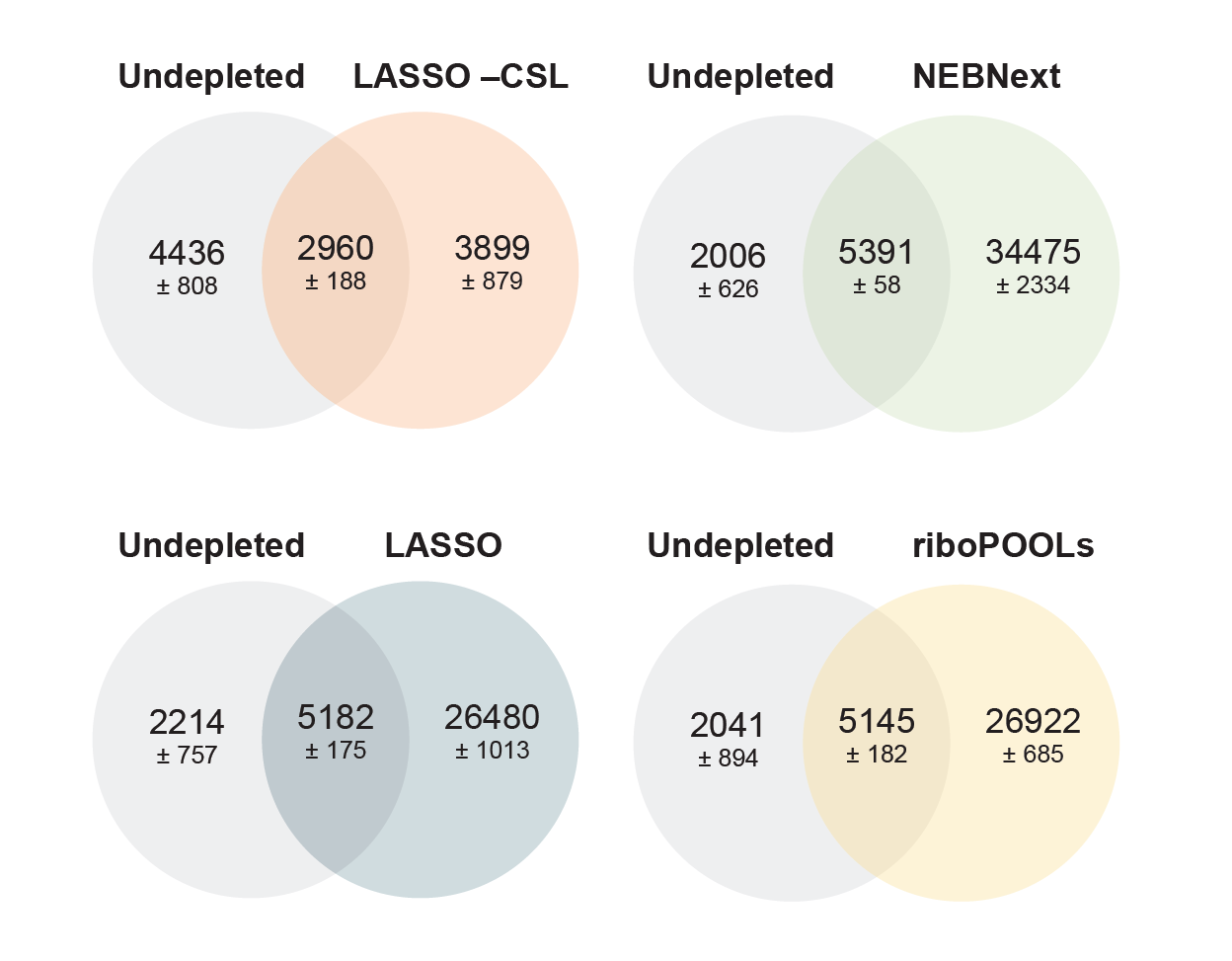


**Figure S11. Comparison of RNA library composition after rRNA depletion.** Venn diagrams for all transcripts with TPM > 1 in rRNA-depleted samples (and the –CSL control) versus the undepleted total RNA sample.


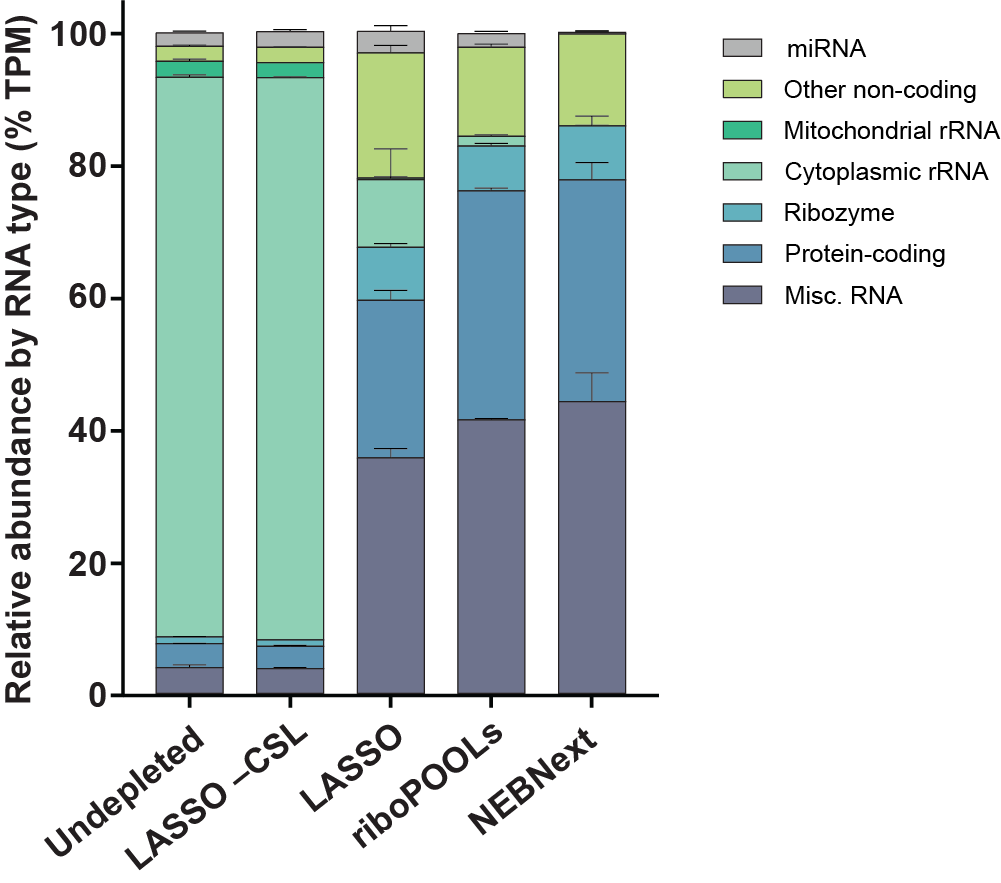


**Figure S12. Biotype distribution including rRNA reads.** All RNA reads were mapped to their respective Ensembl biotype annotation, normalized to transcript per million (TPM), and grouped by type.

**
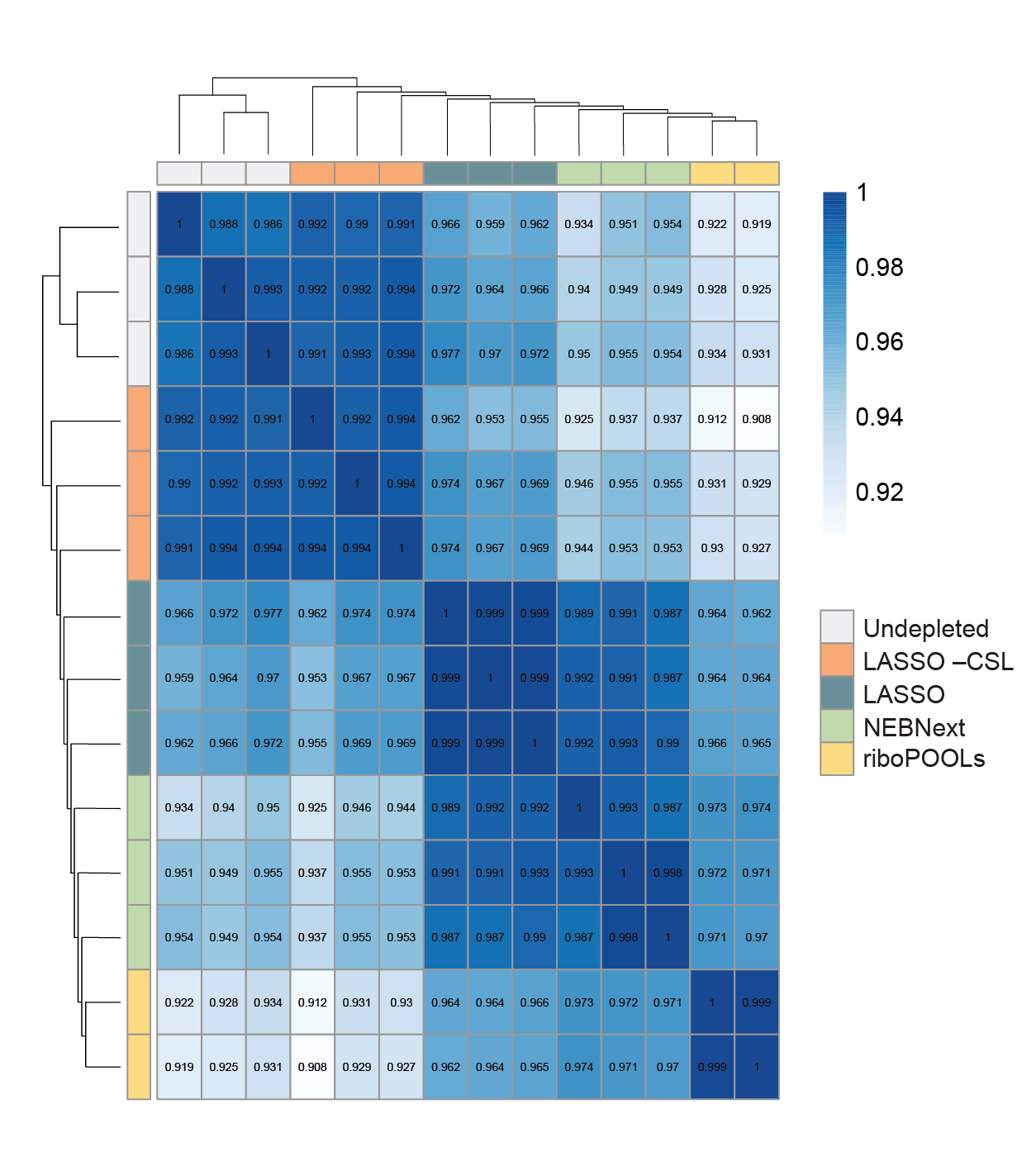
**

**Figure S13. Comparison of Pearson correlation coefficients between methods for rRNA depletion (excluding rRNA depletion targets).**

**
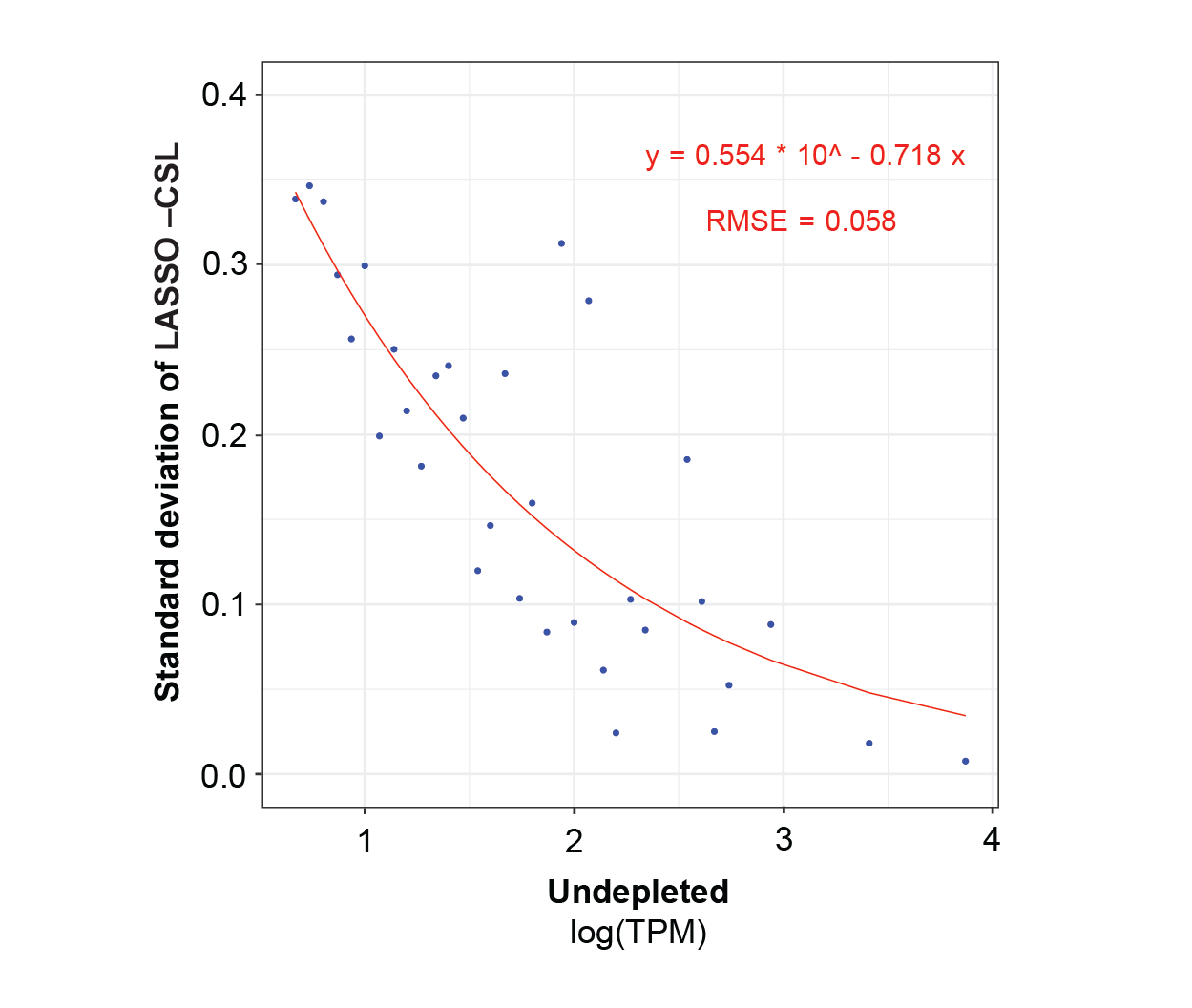
**

**Figure S14. Modeling of expected standard deviations for rRNA-depleted samples.** Outliers in Figure 3d were determined using the exponential model derived in this plot. See Methods section “RNA sequencing (RNA-seq)” for details.

**
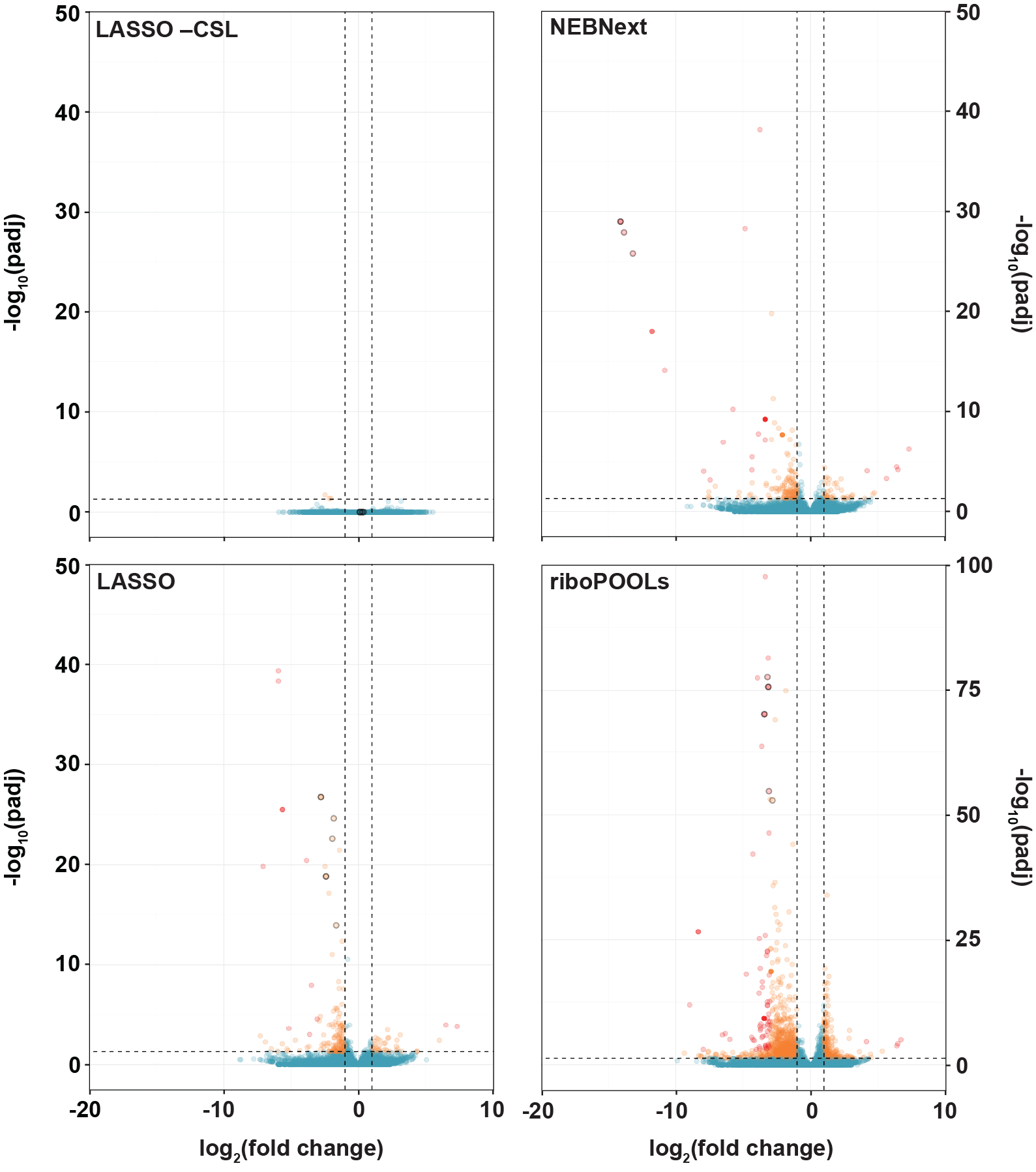
**

**Figure S15. Differential transcript detection from RNA-seq.** Volcano plots showing log₂ fold changes in **detected transcript abundance** between rRNA depletion methods, excluding rRNA transcripts. Transcripts with padj < 0.05 and absolute log₂ fold change > 1 (orange) or padj < 0.001 and absolute log₂ fold change > 3 (red) are considered significantly affected or extremely affected, respectively. Datapoints with black outlines indicate transcripts with high sequence similarity (>90%) to rRNA and are identical to the outlined datapoints in Figure 3d.


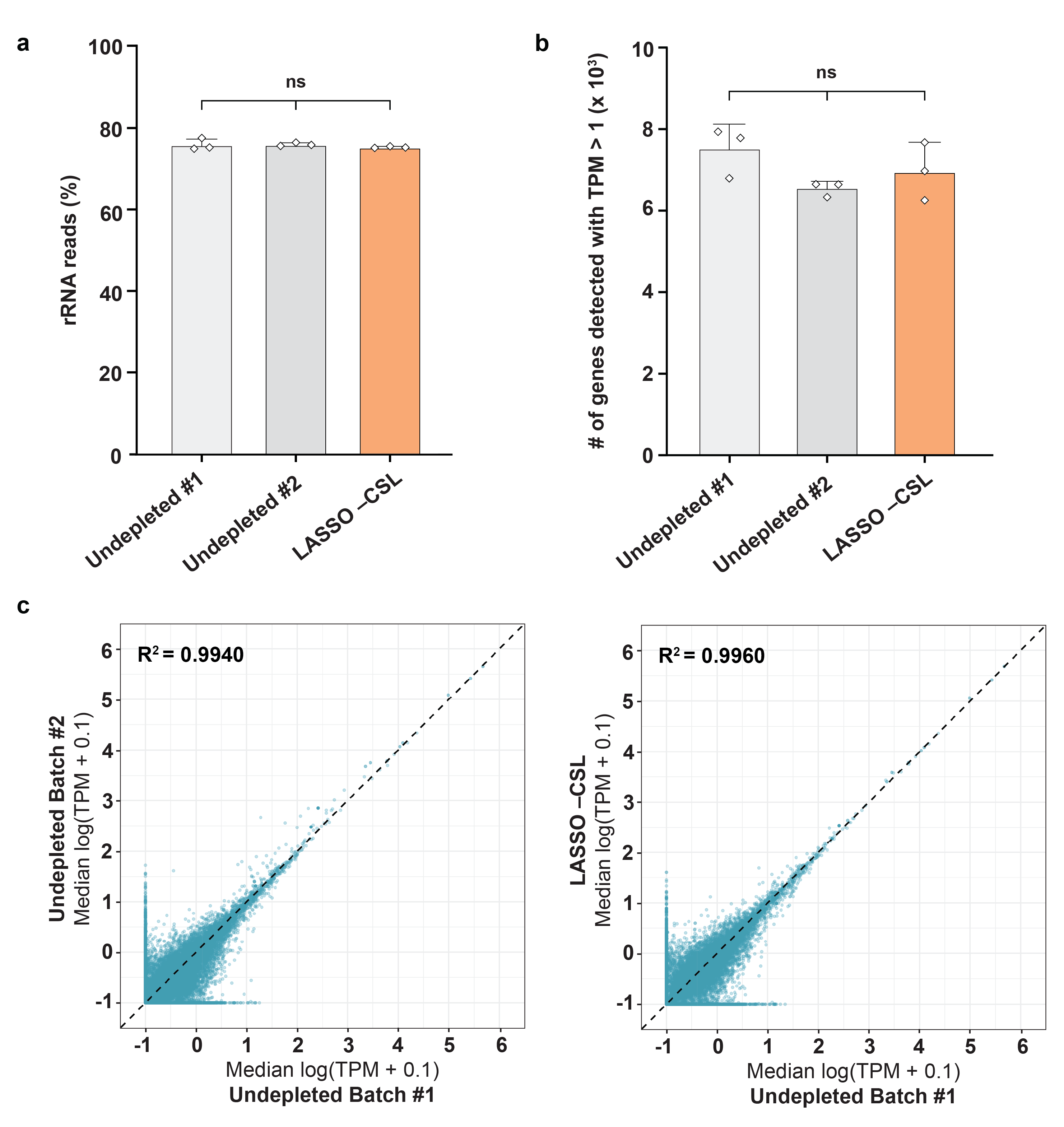


**Figure S16. Comparison between undepleted library replicates and LASSO –CSL indicates near-zero background binding. a)** Percentage of RNA-seq reads mapped to rRNA for each condition. **b)** Total number of transcripts detected with >1 TPM for each condition. **c)** Correlation analysis of expression profiles. Undepleted batches were sequenced independently in triplicate. Statistical analysis for panels a and b was performed via an unpaired two-tailed t-test. ns, non-significant (p > 0.05).

**
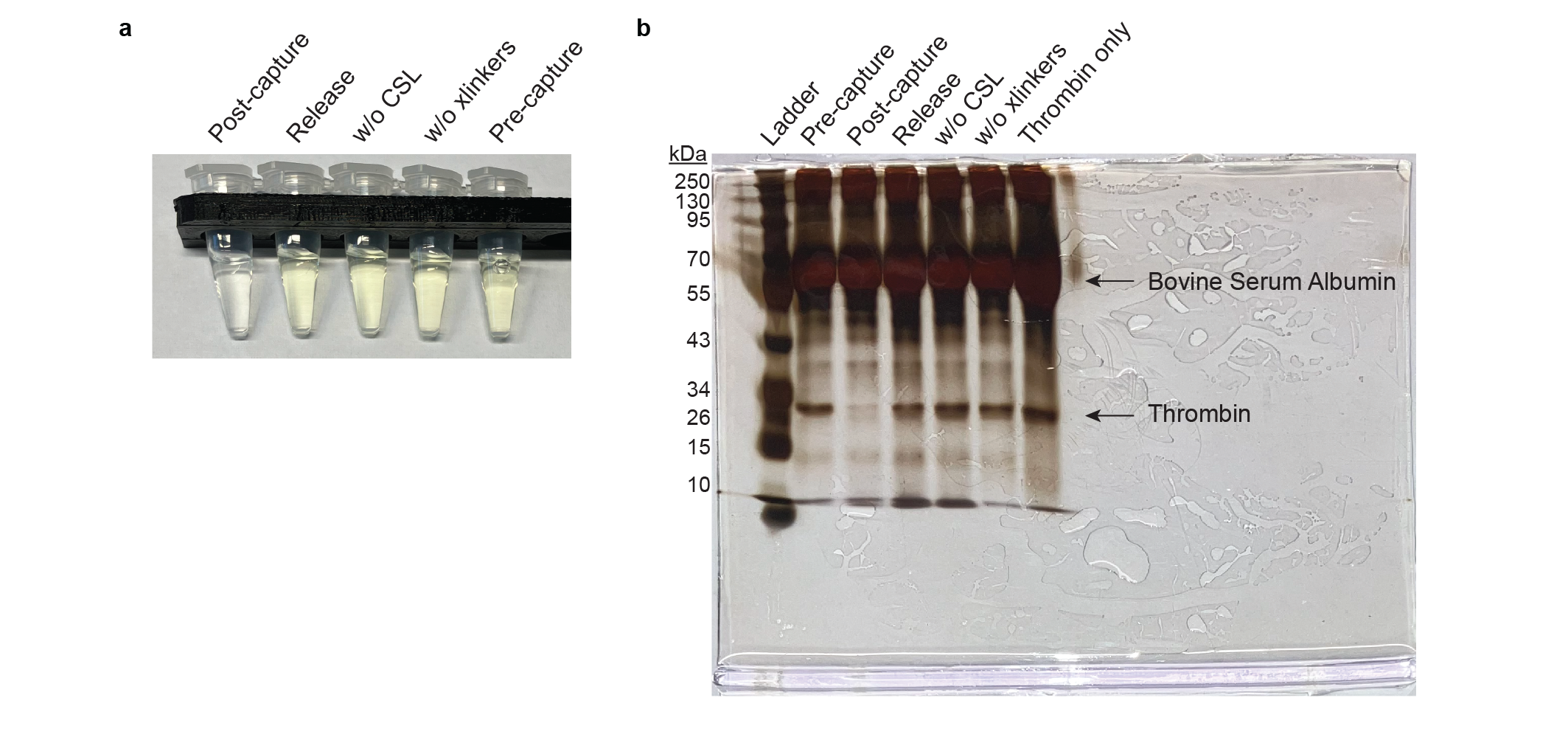
**

**Figure S17**. **Raw images for thrombin capture and release on LASSO. a)** Raw image of tubes containing supernatant samples after cleavage of S-2238 substrate by thrombin. Samples were incubated for 20 minutes at 37°C. **b)** Raw image of supernatant samples on silver-stained 12.5% SDS-PAGE gel. The buffer contains BSA (66 kDa), a common additive for protein stabilization. For details, see Supporting Procedure 4.


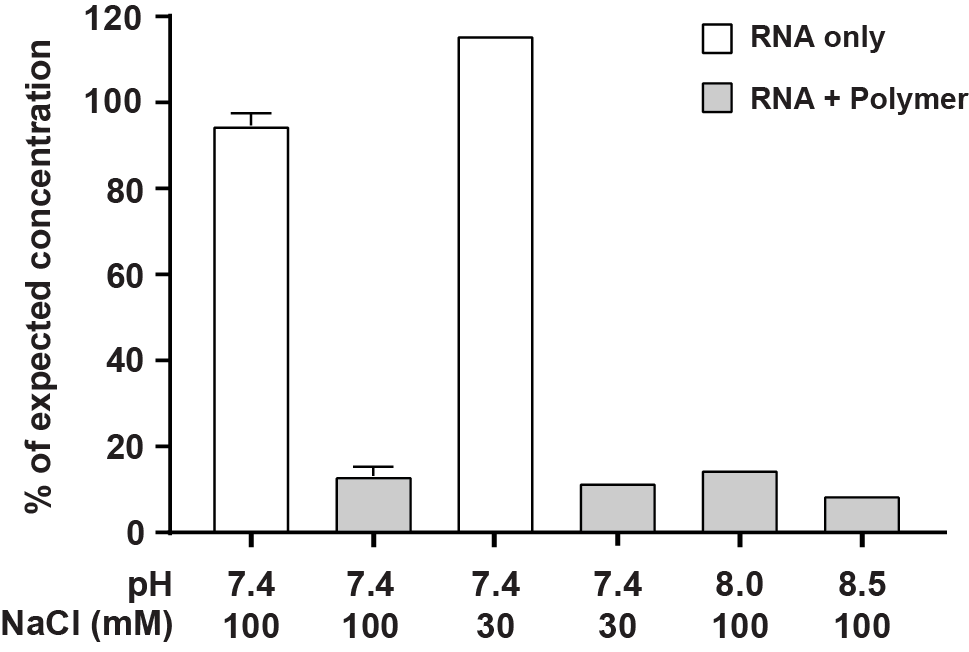


**Figure S18. Nonspecific RNA adsorption on DNA-grafted poly(acrylamide-coacrylic acid) during methanol precipitation.** n = 2 for samples at pH 7.4, 100 mM NaCl; n = 1 for all other samples.


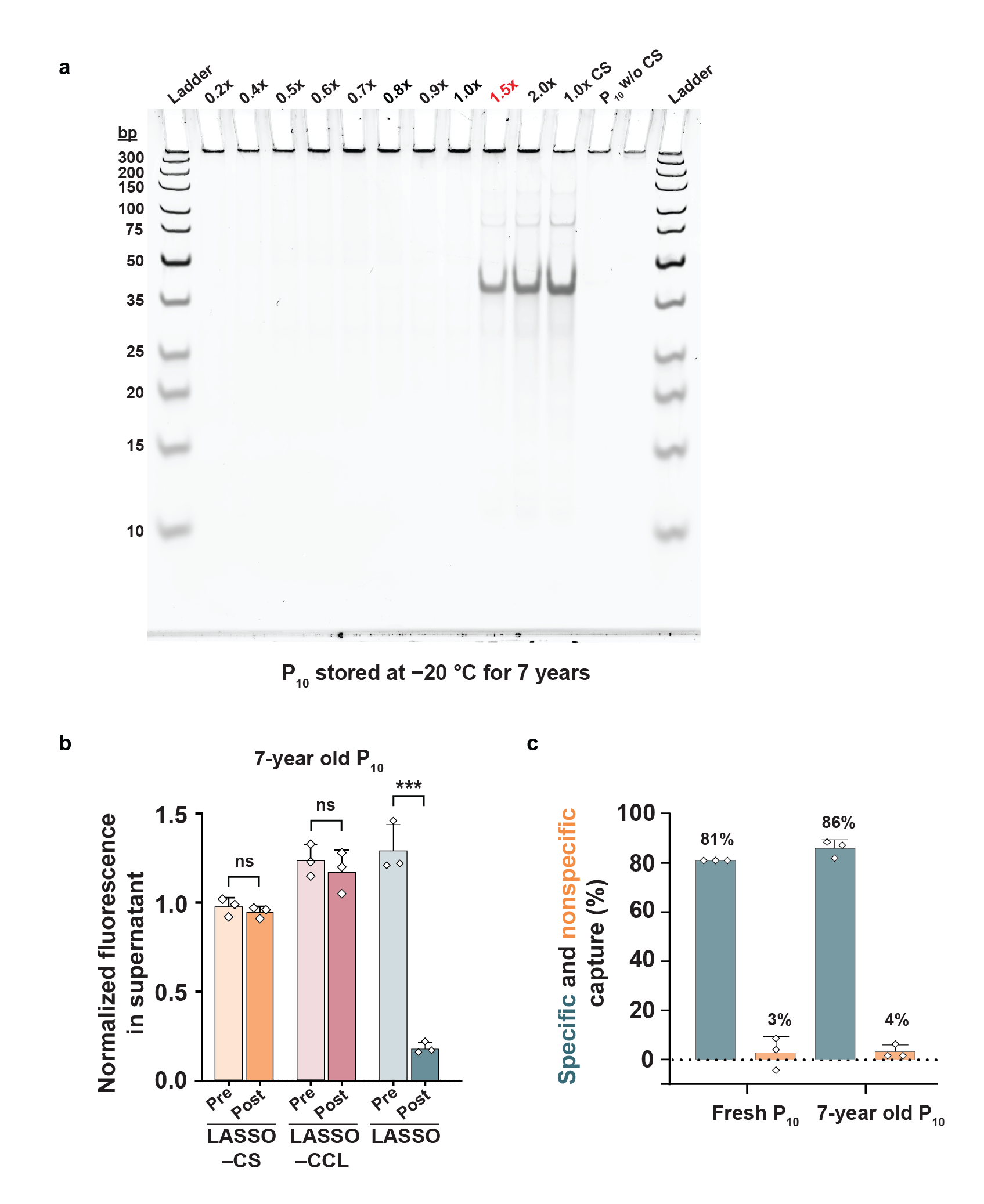


**Figure S19. Long-term stability test of P_10_.** **a)** The polymer had been stored at −20 *°C for seven years. The original concentration of anchor strands after synthesis was quantified as 75 µM for a 0.5% (w/v) stock solution. The polymer was mixed with catcher strands (CS) complementary to the anchor strand at a concentration range from 0.2x to 2.0x, where 1x is defined as 100% of the original binding capacity.* **P_10_** saturated with catcher strands (CS) at 1.5x, indicating the available concentration of anchor strands was approximately 75 µM in a 0.5% (w/v) stock solution after long-term storage, or 100% of the original binding capacity. **b)** The same polymer was used in a LASSO pulldown against a fluorescent ssDNA target. Quantification of the DNA target remaining in the supernatant after pulldown is based on fluorescence intensity. **c)** Comparison of DNA capture efficiency and specificity between fresh **P_10_** and 7-year old **P_10_**. Nonspecific capture was measured from samples subjected to LASSO pulldown without CSL under otherwise identical conditions. Data for panels b and c are shown as mean ± s.d. (n = 3 independent experiments). Statistical analysis was performed using an unpaired two-tailed t-test; ns, non-significant (p > 0.05); ***p < 0.001.

# Supporting Tables

**Table S1.** **Comparison between different smart polymer-based bioseparation methods.**

| **Approach** | **Trigger** | **Key challenges** | | | | **Notes** |
| --- | --- | --- | --- | --- | --- | --- |
|  |  | 1. Risk of target degradation | 2. Risk of premature pulldown | 3. Non-specific co-precipitation | 4. Generally applicable |  |
| PNIPAM^[12–14]^ | **Heat** and/or **salt** | **High** for proteins, **moderate** for nucleic acids | **High** for nucleic acids, **low** for proteins | **Moderate–high** (e.g. 7% for DNA,^[12]^ 20% for proteins^[15]^) | **No**  (proof-of-concept stage, few targets reported) | Scalable, low-cost, risk of target denaturation and off-target capture |
| MeRPy^[16–18]^ | **MeOH** | **High** for proteins, **low** for DNA/RNA | **None**  (MeOH needed) | **Low** for DNA, **high** for RNA and proteins | **No**  (DNA only) | Scalable, low-cost, limited to single- or double-stranded DNA targets |
| ELP^[19–21]^ | **Heat**, **salt**, or **pH** | **Low**  (narrow temp. window) | **Low**  (for most proteins) | **Low–moderate**  (depends on lysate composition) | **No**  (fusion tags necessary) | Scalable, limited to recombinant fusion proteins |
| LASSO  (this work) | **CCL** | **None**  (unchanged physicoche-mical conditions) | **None**  (capture in free solution) | **Low**  (near-zero background binding) | **Yes**  (DNA, RNA, native proteins) | Scalable, gentle capture and release, highly selective, versatile, low-cost |

PNIPAM = poly(N-isopropylacrylamide), MeRPy = methanol-responsive polymer, ELP = elastin-like polypeptide, LASSO = crosslink-assisted sequence-selective isolation.

**Table S2.** **Cost calculation of LASSO.** The cost is calculated per pmol target for general capture and release (for example, a single nucleic acid sequence or protein) and for rRNA depletion using a full CSL on 1 µg of total RNA. The purchase price per reaction for the two commercial rRNA depletion kits is also given.

| **Materials/Reagents** | **Unit** | **Unit cost** | **Cost for general capture and release** | **Cost for rRNA depletion** |
| --- | --- | --- | --- | --- |
| Anchor strand | 1 nmol | $ 0.07 | $ 0.02 | $ 0.35 |
| DNA crosslinkers | 1 nmol | $ 0.09 | $ 0.02 | $ 0.36 |
| DNA catcher strands | 1 nmol | $ 0.15/$ 0.36 | $ 0.00 | $ 0.11 |
| DNA release strands | 1 nmol | $ 0.10 | $ 0.00 | - |
| Hybridization buffer | 1 mL | $ 0.06 | $ 0.00 | $ 0.00 |
| DNase I | 1 unit | $ 0.07 | - | $ 0.14 |
| **Total cost for LASSO pulldown assay** | | | **~ $ 0.04** | **~ $** **0.96** |
| **Cost for NEBNext rRNA depletion kit v2** | | | **-** | **~ $ 51.00** |
| **Cost for riboPOOLs rRNA depletion kit** | | | **-** | **~ $ 46.00** |

**Table S3. Comparison between LASSO and commercial kits for rRNA depletion.**

| **Protocol** | **Protocol Time*** | **Pros** | **Cons** |
| --- | --- | --- | --- |
| LASSO | 1 hour | - Minimal bias and off-target capture - Easy to use (mix and spin) - Inexpensive and highly scalable - Long shelf life | - Moderate depletion efficiency   (~86%) |
| riboPOOLs | 1 hour | - High depletion efficiency (~97%) - Easy to use (mix and pull) - Automation-friendly | - Introduces substantial bias - Expensive |
| NEBNext | 1.5 hours | - Exceptional depletion efficiency (>99%) | - Introduces some bias - Short shelf life - Expensive - Multi-step protocol |

*Net time of the depletion step, not including any additional purification (for example, with SPRI beads or a silica spin column).

# Supporting References

[1] E. Krieg, K. Gupta, A. Dahl, M. Lesche, S. Boye, A. Lederer, W. M. Shih, “A smart polymer for sequence-selective binding, pulldown, and release of DNA targets” *Commun. Biol.* **2020**, *3*, 1–9.

[2] L. R. G. Treloar, *The Physics of Rubber Elasticity*, Oxford University Press, Oxford, New York, **1976**.

[3] K. Gupta, E. Krieg, “Y-switch: a spring-loaded synthetic gene switch for robust DNA/RNA signal amplification and detection” *Nucleic Acids Res.* **2024**, *52*, e80.

[4] X. Adiconis, D. Borges-Rivera, R. Satija, D. S. DeLuca, M. A. Busby, A. M. Berlin, A. Sivachenko, D. A. Thompson, A. Wysoker, T. Fennell, A. Gnirke, N. Pochet, A. Regev, J. Z. Levin, “Comparative analysis of RNA sequencing methods for degraded or low-input samples” *Nat. Methods* **2013**, *10*, 623–629.

[5] N. L. Bray, H. Pimentel, P. Melsted, L. Pachter, “Near-optimal probabilistic RNA-seq quantification” *Nat. Biotechnol.* **2016**, *34*, 525–527.

[6] P. A. Ewels, A. Peltzer, S. Fillinger, H. Patel, J. Alneberg, A. Wilm, M. U. Garcia, P. Di Tommaso, S. Nahnsen, “The nf-core framework for community-curated bioinformatics pipelines” *Nat. Biotechnol.* **2020**, *38*, 276–278.

[7] P. W. Harrison, M. R. Amode, O. Austine-Orimoloye, A. G. Azov, M. Barba, I. Barnes, A. Becker, R. Bennett, A. Berry, J. Bhai, S. K. Bhurji, S. Boddu, P. R. Branco Lins, L. Brooks, S. B. Ramaraju, L. I. Campbell, M. C. Martinez, M. Charkhchi, K. Chougule, A. Cockburn, C. Davidson, N. H. De Silva, K. Dodiya, S. Donaldson, B. El Houdaigui, T. E. Naboulsi, R. Fatima, C. G. Giron, T. Genez, D. Grigoriadis, G. S. Ghattaoraya, J. G. Martinez, T. A. Gurbich, M. Hardy, Z. Hollis, T. Hourlier, T. Hunt, M. Kay, V. Kaykala, T. Le, D. Lemos, D. Lodha, D. Marques-Coelho, G. Maslen, G. A. Merino, L. P. Mirabueno, A. Mushtaq, S. N. Hossain, D. N. Ogeh, M. P. Sakthivel, A. Parker, M. Perry, I. Piližota, D. Poppleton, I. Prosovetskaia, S. Raj, J. G. Pérez-Silva, A. I. A. Salam, S. Saraf, N. Saraiva-Agostinho, D. Sheppard, S. Sinha, B. Sipos, V. Sitnik, W. Stark, E. Steed, M.-M. Suner, L. Surapaneni, K. Sutinen, F. F. Tricomi, D. Urbina-Gómez, A. Veidenberg, T. A. Walsh, D. Ware, E. Wass, N. L. Willhoft, J. Allen, J. Alvarez-Jarreta, M. Chakiachvili, B. Flint, S. Giorgetti, L. Haggerty, G. R. Ilsley, J. Keatley, J. E. Loveland, B. Moore, J. M. Mudge, G. Naamati, J. Tate, S. J. Trevanion, A. Winterbottom, A. Frankish, S. E. Hunt, F. Cunningham, S. Dyer, R. D. Finn, F. J. Martin, A. D. Yates, “Ensembl 2024” *Nucleic Acids Res.* **2024**, *52*, D891–D899.

[8] B. J. Raney, G. P. Barber, A. Benet-Pagès, J. Casper, H. Clawson, M. S. Cline, M. Diekhans, C. Fischer, J. Navarro Gonzalez, G. Hickey, A. S. Hinrichs, R. M. Kuhn, B. T. Lee, C. M. Lee, P. Le Mercier, K. H. Miga, L. R. Nassar, P. Nejad, B. Paten, G. Perez, D. Schmelter, M. L. Speir, B. D. Wick, A. S. Zweig, D. Haussler, W. J. Kent, M. Haeussler, “The UCSC Genome Browser database: 2024 update” *Nucleic Acids Res.* **2023**, *52*, D1082–D1088.

[9] Z.-L. Deng, P. C. Münch, R. Mreches, A. C. McHardy, “Rapid and accurate identification of ribosomal RNA sequences via deep learning” *Nucleic Acids Res.* **2022**, *50*, e60.

[10] M. I. Love, W. Huber, S. Anders, “Moderated estimation of fold change and dispersion for RNA-seq data with DESeq2” *Genome Biol.* **2014**, *15*, 550.

[11] Y.-H. Peng, S.-K. Hsiao, K. Gupta, A. Ruland, G. K. Auernhammer, M. F. Maitz, S. Boye, J. Lattner, C. Gerri, A. Honigmann, C. Werner, E. Krieg, “Dynamic matrices with DNA-encoded viscoelasticity for cell and organoid culture” *Nat. Nanotechnol.* **2023**, *18*, 1463–1473.

[12] M. D. Costioli, I. Fisch, F. Garret‐Flaudy, F. Hilbrig, R. Freitag, “DNA purification by triple-helix affinity precipitation” *Biotechnol. Bioeng.* **2003**, *81*, 535–545.

[13] H. G. Schild, “Poly(N-isopropylacrylamide): experiment, theory and application” *Prog. Polym. Sci.* **1992**, *17*, 163–249.

[14] T. Mori, D. Umeno, M. Maeda, “Sequence-specific affinity precipitation of oligonucleotide using poly(N-isopropylacrylamide)–oligonucleotide conjugate” *Biotechnol. Bioeng.* **2001**, *72*, 261–268.

[15] S. Suzuki, T. Sawada, T. Ishizone, T. Serizawa, “Affinity-based thermoresponsive precipitation of proteins modified with polymer-binding peptides” *Chem. Commun.* **2016**, *52*, 5670–5673.

[16] E. Krieg, K. Gupta, A. Dahl, M. Lesche, S. Boye, A. Lederer, W. M. Shih, “A smart polymer for sequence-selective binding, pulldown, and release of DNA targets” *Commun. Biol.* **2020**, *3*, 369.

[17] D. Minev, R. Guerra, J. Y. Kishi, C. Smith, E. Krieg, K. Said, A. Hornick, H. M. Sasaki, G. Filsinger, B. J. Beliveau, P. Yin, G. M. Church, W. M. Shih, “Rapid in vitro production of single-stranded DNA” *Nucleic Acids Res.* **2019**, *47*, 11956–11962.

[18] E. Krieg, W. M. Shih, “Selective Nascent Polymer Catch-and-Release Enables Scalable Isolation of Multi-Kilobase Single-Stranded DNA” *Angew. Chem. Int. Ed.* **2018**, *57*, 714–718.

[19] T. Díez Pérez, A. N. Tafoya, D. S. Peabody, M. R. Lakin, I. Hurwitz, N. J. Carroll, G. P. López, “Isolation of nucleic acids using liquid–liquid phase separation of pH-sensitive elastin-like polypeptides” *Sci. Rep.* **2024**, *14*, 10157.

[20] W. Hassouneh, T. Christensen, A. Chilkoti, “Elastin-Like Polypeptides as a Purification Tag for Recombinant Proteins” *Curr. Protoc. Protein Sci.* **2010**, *61*, 6.11.1-6.11.16.

[21] D. E. Meyer, A. Chilkoti, “Purification of recombinant proteins by fusion with thermally-responsive polypeptides” *Nat. Biotechnol.* **1999**, *17*, 1112–1115.
